# Discrimination sensitivity of visual shapes sharpens in autistic adults but only after explicit category learning

**DOI:** 10.1186/s13229-024-00604-6

**Published:** 2024-06-03

**Authors:** Jaana Van Overwalle, Birte Geusens, Stephanie Van der Donck, Bart Boets, Johan Wagemans

**Affiliations:** 1 Department of Brain and Cognition, Leuven Brain Institute, KU Leuven, Leuven, 3000 Belgium; 2 Center for Developmental Psychiatry, Leuven Brain Institute, KU Leuven, Leuven, 3000 Belgium; 3 Leuven Autism Research (LAuRes), KU Leuven, Leuven, 3000 Belgium

**Keywords:** Frequency-tagging electroencephalography, Category learning, Discrimination sensitivity, Visual perception, Autism

## Abstract

**Background:**

Categorization and its influence on perceptual discrimination are essential processes to organize information efficiently. Individuals with Autism Spectrum Condition (ASC) are suggested to display enhanced discrimination on the one hand, but also to experience difficulties with generalization and ignoring irrelevant differences on the other, which underlie categorization. Studies on categorization and discrimination in ASC have mainly focused on one process at a time, however, and typically only used either behavioral or neural measures in isolation. Here, we aim to investigate the interrelationships between these perceptual processes using novel stimuli sampled from a well-controlled artificial stimulus space. In addition, we complement standard behavioral psychophysical tasks with frequency-tagging EEG (FT-EEG) to obtain a direct, non-task related neural index of discrimination and categorization.

**Methods:**

The study was completed by 38 adults with ASC and 38 matched neurotypical (NT) individuals. First, we assessed baseline discrimination sensitivity by administering FT-EEG measures and a complementary behavioral task. Second, participants were trained to categorize the stimuli into two groups. Finally, participants again completed the neural and behavioral discrimination sensitivity measures.

**Results:**

Before training, NT participants immediately revealed a categorical tuning of discrimination, unlike ASC participants who showed largely similar discrimination sensitivity across the stimuli. During training, both autistic and non-autistic participants were able to categorize the stimuli into two groups. However, in the initial training phase, ASC participants were less accurate and showed more variability, as compared to their non-autistic peers. After training, ASC participants showed significantly enhanced neural and behavioral discrimination sensitivity across the category boundary. Behavioral indices of a reduced categorical processing and perception were related to the presence of more severe autistic traits. Bayesian analyses confirmed overall results.

**Limitations:**

Data-collection occurred during the COVID-19 pandemic.

**Conclusions:**

Our behavioral and neural findings indicate that adults with and without ASC are able to categorize highly similar stimuli. However, while categorical tuning of discrimination sensitivity was spontaneously present in the NT group, it only emerged in the autistic group after explicit categorization training. Additionally, during training, adults with autism were slower at category learning. Finally, this multi-level approach sheds light on the mechanisms underlying sensory and information processing issues in ASC.

**Supplementary Information:**

The online version contains supplementary material available at 10.1186/s13229-024-00604-6.

## Background

Autism Spectrum Condition (ASC) is a neurodevelopmental disorder characterized by difficulties in social interaction and communication, and by the presence of restricted, repetitive patterns of behavior, interests or activities [1, 2]. In the latest version of the Diagnostic and Statistical manual of Mental disorders (DSM-5) atypical sensory processing was added as a diagnostic criterion [2]. This was in line with scientific evidence, which has expanded to atypical sensory processing as a key area in ASC [3]. This shift has stimulated new interest in perceptual processes in ASC, such as perceptual discrimination and categorization [4].

### Atypical sensory processing (such as sensory sensitivity) has a huge impact on the daily life of individuals with ASC

For instance, this results in the tendency of autistic individuals to avoid noisy environments. On the other hand, their sensitivity may also contribute to sensory seeking behaviors [5]. These experiences in daily life map well on the proposition that individuals with ASC show enhanced low-level perceptual processing [6]. Indeed, evidence implies that discrimination is enhanced in individuals with ASC [4, 7]. *Perceptual discrimination* is essential to perceive differences and differentiate between different exemplars and situations, but also requires balance with their counterpart categorization. When categorizing, on the other hand, irrelevant differences between stimuli need to be ignored to be able to group them together as a perceptual or cognitive category. *Categorization* allows one to respond quickly and adaptively to new exemplars of a known category, by treating these new exemplars as somehow equivalent [8]. This process is crucial to interact appropriately with the world, in order to avoid being overwhelmed by its complexity and variability. The balance between perceptual discrimination and categorization requires some level of abstraction, (i.e., to extract the relevant similarities in spite of minor or irrelevant differences), but one still needs to be able to discriminate between different exemplars of a known category (e.g., different faces). A well-documented interaction between categorization and discrimination is categorical perception, which is reflected in reduced discrimination sensitivity for exemplars within a category and enhanced discrimination (“discrimination peak”) for exemplars across the category boundary [9]. Evidence suggests that individuals with ASC experience difficulties in categorization [4, 10]. In their recent meta-analysis, Wimmer and colleagues provided evidence for reduced category learning skills in ASC [10]. In addition, Mercado and colleagues discussed how atypical perceptual category learning in ASC can affect their cognitive development and social functioning [11].

### Difficulties in categorization and an enhanced perceptual discrimination in ASC may be in line with different hypotheses concerning learning in autism

For example, hypothesized differences in learning style postulate that autistic individuals’ prefer *look-up table* (LUT) learning, which aims at storing exemplars or experiences precisely, instead of *interpolation* (INT) learning, which focuses on extracting the underlying regularities from experiences [12]. At a neurocognitive level, these perceptual and learning processes can also be linked to the predictive coding [13]. According to this account, the brain is a prediction machine that attempts to match incoming sensory input with top-down expectations or predictions. Depending on the situation, a mismatch (i.e., prediction error) can either be relevant and should be taken into account to update expectations or should be ignored [14]. Van de Cruys and colleagues argued in their HIPPEA account (i.e., high and inflexible precision of prediction errors in autism) that updating of the internal models cannot be flexibly adjusted to the complexity of the environment in individuals with ASC, which gives rise to overfitting of sensory input, resulting in overspecific categories and reduced generalization, and consequently an overwhelming information overload in ASC [15].

### Studies investigating these perceptual processes in ASC are rather limited and conflicting

Previous research focused mostly on the hypothesis that individuals with ASC are not able to categorize stimuli using prototypes (i.e., the most typical exemplars that exhibit the essential features of a category) [17–20]. The results of this research have been conflicting by not taking into account confounding factors (i.e., differences in participant characteristics, experimental paradigm, given instructions, how participants were trained, application of feedback, etc.), by disregarding heterogeneity within group and by using experimental approaches and stimuli that are not always suitable to study prototype formation [16, 21]. In addition, autistic individuals would be slower in learning categories [22], display no typical “discrimination peak” (i.e., no higher discrimination sensitivity across the category compared to within the category; [23]), have difficulties categorizing atypical exemplars [24, 28], are less inclined to use an *interpolation* learning style (i.e., INT: extracting regularities; [12, 25]), have reduced generalization of category learning [26, 27], or would have difficulties with multi-dimensional categorization (i.e., when stimuli differ on two or more dimensions; [29, 30]). Thus far, almost no study has investigated the interplay between perceptual discrimination and category learning, to address sensory and information processing issues in ASC and the underlying mechanisms in a coherent way [18, 24, 31]. Furthermore, most studies investigating perceptual discrimination and categorization in individuals with and without ASC were limited to behavior. A particular disadvantage of the exclusive use of behavioral tests is that responses are not controlled for various cognitive processes and biases, such as decisional or motivational processes, which could obscure differences between participants with and without ASC.

### Opportunities for innovative direct neural measures

Against this background, a more direct neural index of perceptual discrimination and categorization is needed. We believe that Frequency-Tagging (FT) scalp electroencephalography (EEG) recording during periodic stimulation provides exactly that. The principle of FT-EEG refers to visual stimulation of the human brain at a constant frequency rate (e.g., 6 Hz), which evokes an EEG response on the scalp exactly at that frequency (i.e., steady-state visual evoked potential or SSVEP) [32]. The application of the FT-EEG in an oddball paradigm, that is, the detection of periodically introduced oddball (O) images in a series of base (B) images (e.g., B, B, B, B, O, B) by an EEG response at the oddball frequency (e.g., 1.2 Hz), makes it an objective and direct measure for change detection [33]. The FT-EEG approach has many advantages: (1) the response can be measured automatically (i.e., spontaneously, without an explicit behavioral task)^1^ and objectively (since it occurs at a predefined frequency); (2) the response can be quantified directly by comparing the response at the induced frequency (signal) with responses at neighboring frequencies (noise); (3) the technique is extremely robust, since it yields high signal-to-noise ratio (SNR) responses; and (4) the technique provides these robust results in a few minutes only. FT-EEG paradigms have been validated in the context of low-level and mid-level visual processing (e.g., contrast sensitivity and figure-ground segregation) [32] as well as higher level face processing (e.g., face discrimination) [33, 34]. In a recent paper with NT participants, we also showed that FT-EEG can provide a direct neural index of perceptual discrimination and categorization [35].

### The present study

The ability to categorize is an essential cognitive function which may be impaired in ASC. As argued earlier, this difficulty relates to differences in perceptual discrimination. It is probably due to differences in learning style, and more specifically differences in error correction. However, earlier research on perceptual discrimination and categorization mostly happened in isolation, using rather few trials and naturalistic stimuli, with poorly controlled dimensions or features [11]. The present study aims to provide an in-depth understanding by investigating the processes of discrimination and categorization concurrently. By using a systematic approach, combining solid psychophysical paradigms and a carefully controlled stimulus set with FT-EEG measures, we will circumvent the problems of earlier research and attempt to draw clearer conclusions.

In particular, Soulières and colleagues argued that individuals with ASC are able to categorize, but are slower in learning this process [22]. In another study, they found that individuals with ASC do not show the typical behavioral “discrimination peak” of categorical perception [23]. However, their findings are not unequivocal, as their stimuli (i.e., ellipses) may not have induced a categorical percept since their experiment did not involve a distinct pre- and post-categorization training measurement. To overcome these ambiguities, we will investigate potential shifts in discrimination sensitivity (across the to-be trained category boundary compared to within the category) before and after explicit categorization training (via feedback) in neurotypical (NT) and ASC participants on two highly-controlled orthogonal stimulus dimensions (counterbalanced across participants). Using two orthogonal dimensions enables us to draw general conclusions about the induced categorization training effects, which are not stimulus specific. Moreover, training occurred on different exemplars from the original dimension, forcing the induction of an *interpolation* learning-style (not an exemplar-based LUT style). Finally, in this study, we will combine direct FT-EEG measures, together with standard psychophysical behavioral tasks, to get a more complete impression of discrimination sensitivity before and after category learning.

## Methods

### Participants

Seventy-six participants (38 with and 38 without an ASC diagnosis) took part in the study. Participants were normally gifted adults (18–55 year age range, IQ > 75) with corrected-to-normal vision, without neuroleptics prescription and without prior knowledge about the experiment. Participants were recruited through public advertisement and the Leuven Autism Expertise Center. Participants with ASC were well-phenotyped and diagnosed by a multidisciplinary team following DSM-IV or DSM-5 criteria. NT and ASC participants were matched on gender, age, laterality, and IQ (Table 1). For NT participants, additional exclusion criteria were being diagnosed with a psychiatric or neurological disorder, and an Autistic-spectrum Quotient (AQ) above the clinical cut-off of 32. In the ASC group, fourteen participants reported having one or several comorbidities (attention-deficit (hyperactivity) disorder: 5, dyslexia, dyscalculia and/or dyspraxia: 3, combination of attention-deficit hyperactivity disorder and dyslexia/depression: 2, depression: 1, obsessive-compulsive disorder: 1, Gilles de la Tourette: 1, epilepsy: 1).

**Table 1:** Participants’ demographics and questionnaire scores. Group means (± standard deviations). Results of t-tests and chi-square test: ns: non-significant (*p* > 0.05). Note that GSQ questionnaire scores from one ASC participant were missing

	NT group	ASC group	*p*
Number of participants	38	38	-
Male / Female number	20/18	21/17	ns (*p* = 0.77)
Age (years)	29.0 (± 8.1)	32.6 (± 9.3)	ns (*p* = 0.07)
Left / Right-handed	5/33	7/31	ns (*p* = 0.61)
Intelligence Quotient (FSIQ)	112.4 (± 13.3)	107.3 (± 16.4)	ns (*p* = 0.14)
VBI	109.5 (± 13.4)	107.2 (± 14.6)	ns (*p* = 0.46)
PRI	109.6 (± 15.4)	104.8 (± 18.2)	ns (*p* = 0.21)
Autism-spectrum Quotient	11.6 (± 5.5)	30.4 (± 9.0)	*p* < 0.0001
Glasgow Sensory Sensitivity Scale	28.7 (± 15.1)	55.5 (± 26.6)	*p* < 0.0001

A short version of the Wechsler Adult Intelligence Scale IV-NL (WAIS-IV-NL) was used to estimate IQ of the participants. The four subtests included two tests for verbal comprehension (similarities of words and vocabulary) and two for perceptual reasoning (block design test and visual puzzles). In addition, participants also had to complete an online web survey (via a platform of the KU Leuven) asking about demographics and several personality characteristics related to ASC. The following questionnaires were included: Autism Spectrum Quotient (AQ) and the Glasgow Sensory Sensitivity Scale (GSQ). The AQ is used to assess autistic traits [36, 37]. The GSQ is a 42-item questionnaire assessing atypical sensory sensitivity across seven modalities (i.e., visual, auditory, olfactory, gustatory, tactile, vestibular and proprioceptive [38, 39]). The GSQ gives a total score of atypical sensory sensitivity (maximum score of 168), as well as the separate sub-scores of hypersensitivity and hyposensitivity (with both a maximum score of 84).

The study was approved by the Ethical Committee of the University Hospital of Leuven. Participants provided written informed consent before the start of the experiment and received a monetary compensation afterwards. Participants were instructed to be well-rested to optimize the conditions of the study. The participants were informed about the appearance of flickering images, the duration of the experiments and the breaks. They were asked to complete their task as well as possible and to stay attentive. During the EEG measurements they were instructed to move as little as possible, to minimize their muscle contraction.

### Apparatus and data acquisition

The study was performed in a quiet room where light and environmental sounds were reduced. The experiments were programmed in Psychopy2 [40]. Stimuli were presented on a gray background of a 27-inch LCD monitor with a screen resolution of 2560 × 1440 pixels, and 60 Hz refresh rate. Participants were positioned at a distance of 80 cm using a chin rest.

EEG was recorded using a BioSemi Active-Two amplifier system with 64 Ag/AgCl electrodes. During recording, the system used two additional electrodes for reference and ground (CMS, common mode sense, and DRL, driven right leg). Horizontal and vertical eye movements were recorded using four electrodes placed at the outer canthi of the eyes and above and below the right orbit. The EEG was sampled at 512 Hz and electrode impedances were kept above -30 µV and under 30 µV.

### General procedure

**Fig. 1:**
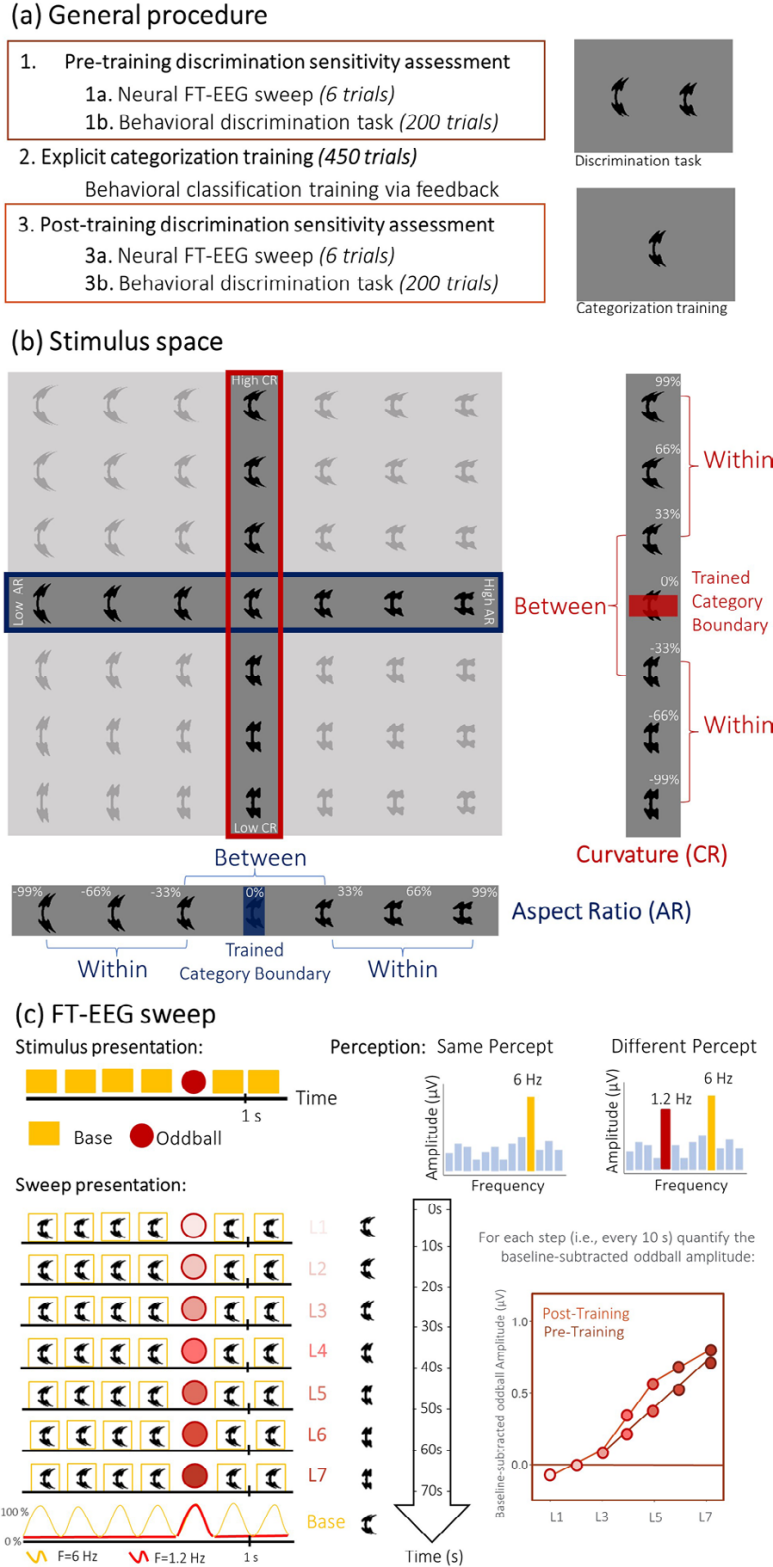
General procedure, stimuli and FT-EEG paradigm. **(a)** Participants’ neural and behavioral discrimination sensitivity was assessed before and after explicit categorization training. **(b)** Stimuli (used for this assessment) consisted out of shapes varying across seven steps along one (i.e., the assigned) of two dimensions: aspect-ratio (AR i.e., the width-to-height ratio) and curvature (CR i.e., the degree to which a curve deviates from a straight line). The category boundary for the assigned dimension was introduced at the midpoint of the dimension (i.e., 0%: fourth stimulus) during the explicit categorization training. To look at behavioral discrimination sensitivity across the assigned stimulus dimension, we compared perceptual discrimination of stimulus pairs within (i.e., pair 1–3 and pair 5–7) and between the trained category (i.e., pair 3–5). **(c)** For the neural discrimination sensitivity assessment, we used an FT-EEG sweep paradigm, which was swept for the oddball stimuli across the assigned stimulus dimension (e.g., curvature) while the base stimulus stayed the same (i.e., one of the end-points, in this example is the 99% curved). To compare neural discrimination sensitivity along the stimulus dimension, we specifically looked at the FT-EEG baseline-subtracted oddball amplitude of stimuli at sweep step 2 and 6 to assess neural sensitivity within the category and of stimuli at sweep step 4 for neural sensitivity across the trained category boundary

Participants performed one study session at the University Hospital of Leuven. During the session, we investigated categorization training and discrimination sensitivity before and after the categorization training with psychophysical and FT-EEG neural measures [35]. First, as a baseline before categorization training, neural measures via FT-EEG sweep were obtained (15’), after which participants performed a behavioral discrimination task (15’). After categorization training (30’), the same FT-EEG measures (15’) and the behavioral discrimination task (15’) were applied again. Identical measures were administered during the pre- and post-assessment (see Fig. 1A).

Participants were trained and assessed on one of two orthogonal dimensions (originally from a two-dimensional stimulus space^2^, see Fig. 1B). Counterbalancing the assigned stimulus dimension across participants (50%-50%) enables us to draw more general conclusions about categorization training effects which are not stimulus specific. Evidently, the assignment of the stimulus dimension that had to be trained and assessed was also matched between ASC and NT groups, while also controlling for participant characteristics as gender, age, laterality, and IQ (see Supplementary Material Table S1). We also examined possible differences elicited by the different stimulus dimensions.

### Stimuli

The stimuli used in this study originate from Ons et al. [41]. These researchers created a highly controlled artificial multi-dimensional stimulus space, consisting of shapes varying along two dimensions: aspect-ratio (AR; i.e., the width-to-height ratio) and curvature (CR; i.e., the degree to which a curve deviates from a straight line). In our study, we used the algorithms of Ons et al. [41] to apply these AR and CR variations to one basic shape for each dimension. For the pre- and post-training assessments, we applied the original seven-step continuum (with steps of 33% from the dimension midpoint to endpoint, see Fig. 1B). For the training session, we used a more fine-grained stimulus space, and applied a 50-step stimulus continuum (with steps of 4%). Based on behavioral pilot experiments, one (unfamiliar) basic artificial shape was selected, and the orthogonal dimensions (CR and AR) were adjusted to equate their respective learning difficulty. Stimuli extended 5 × 2 (vertical x horizontal) visual degrees.

***Pre- and post-training assessment*** To investigate discrimination sensitivity across the assigned stimulus dimension, we compared perceptual discrimination of stimulus pairs within and between the trained category (see Fig. 1B). This assessment happened both before and after undergoing the categorization training, and entailed both behavioral and neural measures.

***Categorization training*** The training was conducted on the assigned dimension (either AR or CR). The category boundary was introduced in the middle of the stimulus dimension (see Fig. 1B). For training purposes, we used training stimuli that differed from the stimuli assessed along each dimension (before and after training). This enabled an interpolation (INT) learning-style instead of an exemplar-based (LUT) learning-style, because participants were not trained and assessed on the same stimuli exemplars [12]. Each dimension was cut-up in smaller steps of 4% giving rise to 50 training stimuli Afterwards, for analyses (such as fitting), we post-hoc reduced (i.e., accordingly averaged) the responses to a 10-step stimulus continuum (see Statistical data analysis Sect.).

### Experimental paradigm

In the study, we investigated categorical training and assessed discrimination sensitivity twice, once before and once after categorization training (see Fig. 1A). Here, we describe one such assessment session and the categorization training task.

#### Neural measures: sweep frequency-tagging oddball EEG paradigm

The principle of the FT-EEG oddball paradigm is the detection of periodically introduced oddball (O) images in a series of base (B) images at the base frequency by an EEG response at the oddball frequency (B, B, B, B, O, B), which makes it an objective and direct measure for perceptual discrimination (see Fig. 1C). More specifically, when oddball stimuli are not perceived as different from the base stimuli, the stimulus presentation will only elicit responses at the base frequency (6 Hz). When oddball stimuli are perceived as different from the base stimuli, they will elicit an additional oddball response at 1.2 Hz and harmonics (n x 1.2 Hz, see Fig. 1C). The amplitude of the oddball response thus gives a neural index of discrimination sensitivity. Using size variations and an orthogonal color change detection task, we can control for low-level and attentional confounds.

The neural part of the experiment consisted of six FT-EEG trials of 70 s (for each assessment), sweeping through the stimuli of the assigned dimension (CR or AR), with stimuli starting from each of the end-points of the stimulus dimension (i.e., in case of CR: progressing from curved to less curved and vice versa, and in case of AR: progressing from elongated to compact and vice versa). In a sweep oddball paradigm, the base stimulus remains fixed throughout the entire trial, while the oddball stimulus systematically progresses along the stimuli of the dimension. For instance, at the beginning of the CR sweep trial (see Fig. 1C), the base and oddball stimulus were identical (i.e., 99% curved), and after every 10 s (or 12 presentations of the same base-oddball stimuli combination) the oddball stimulus systematically changed to the next level (i.e., “66% curved”, “33% curved”, etc.), reaching the “-99% less curved” level after 7 steps. Neural discrimination sensitivity across this same dimension was also assessed while sweeping from the opposite direction (i.e., starting with a base and oddball stimulus at -99% (reduced curvature), and systematically changing the oddball stimulus towards more curvature).

For each of the 7 oddball levels (sweep steps), the stimuli were presented for 10 s leading to a total duration of each sweep trial of 70 s with a fade-in and fade-out of 1.67 s (see Movies ‘CR_Sweep’ and ‘AR_Sweep’). Each sweep trial (alternately in the ‘original’ and the reverse direction along the dimension) was presented three times, both before and after training. This enables us to assess neural discrimination sensitivity along the stimulus dimension before and after training by comparing the baseline-subtracted oddball amplitude during FT-EEG assessment before and after training. We will specifically compare the baseline-subtracted oddball amplitude of the FT-EEG sweep steps 2 and 6 to assess neural discrimination sensitivity for within the category and step 4 for neural discrimination sensitivity across the trained category boundary (see Fig. 1C and EEG data analysis Sect. and Statistical data analysis Sect.).

Each sweep trial started with the presentation of a fixation cross (jittered between 2 and 5 s) in the center of the screen, after which the base and oddball stimuli were presented (in the center of the screen with fixation cross) using a sinusoidal contrast modulation at their respective presentation frequency (6 Hz for the base stimuli and 1.2 Hz for the oddball stimuli). Relative size variations of the presented stimuli at 10% [10% smaller, normal size, 10% larger size] were randomly implemented with a different size at every consecutive presentation to abolish the impact of low-level (pixel) confounds. To ensure attention during passive viewing of the stimuli, participants were instructed to perform an orthogonal color change detection task, with the fixation cross changing color 20 times during each trial. After every trial, the participant had a short break of 10 s. One longer and self-paced break was given in the middle of all the FT-EEG trials.

#### Behavioral discrimination task

To assess behavioral discrimination sensitivity along the assigned stimulus dimension, participants performed a 2-Alternative Forced Choice (2-AFC) same-different task. During the task, two stimuli from the dimension appeared simultaneously (left and right) on the screen and participants had to indicate whether the shape was same or different, regardless of the size. Throughout the task, participants were presented with 10 stimulus pairs. These pairs comprised three “different pairs” (i.e., pair 1–3 and pair 5–7 to assess sensitivity within the category, and pair 3–5 to assess sensitivity across the trained category boundary, see Fig. 1B), which were presented with each stimulus either on the left or on the right side of the screen, thus totaling six “different pairs”. In addition, four “same pairs” were presented, in which each stimulus was compared to itself. Each pair was presented 20 times, giving a total of 200 trials which were organized in four blocks of each 50 trials (corresponding to five presentations of each pair). The trial order was pseudorandomized to prevent consecutive presentation of identical trials. One break was included throughout the task. To ensure that participants understood the task, they performed 10 practice trials on a similar dimension of another stimulus set (with another shape as basis, see Ons et al. [41]).

A trial consisted of a fixation cross for 1 s, a target stimulus pair for 200 ms, and the mask stimuli (i.e., a random squared grayscale pattern, independent of the stimulus properties) for 200 ms to avoid after-image effects. In addition, like the FT-EEG experiment, the relative size of the stimuli varied by 10% [10% smaller, normal size, 10% larger] with a different size at every consecutive presentation. Participants were instructed to respond as fast and as accurately as possible after stimulus presentation started (maximum time limit of 10 s). With keys 1 and 3 on the numerical keypath, counterbalanced across participants, participants had to indicate whether the stimuli were same or different. No direct feedback on the performance was offered, but change in color (e.g., green) of the labels indicated a registered response.

#### Categorization training task

During explicit categorization training, participants were requested to assign a training stimulus correctly to one of two (arbitrary) categories. No direct instructions were offered but participants received explicit feedback on every trial to derive the underlying categories. We administered three training blocks with increasing difficulty level, i.e., by increasing the number of stimuli near the category boundary. In the first block, the 50 training stimuli (see Stimuli Sect.) were each shown three times. In the second block, the percentage of exemplars shown around the category boundary relative to the more typical category exemplars was 60%-40%. In the third block, this balance increased to 80%-20%. In total, participants were trained on 450 trials with 150 trials per block (30’). In each block, presentation of the stimuli occurred in a (pseudo)random order. The order was pseudorandomized to prevent consecutive presentation of identical stimuli. A break was included for each participant at the end and in the middle of each block (i.e., after 75 trials). During each of these self-paced breaks, participants could see their accuracy percentage on the last 75 trials to motivate them to reach a high accuracy.

The structure of a trial was identical to the discrimination task, except that only one stimulus and afterwards mask was presented in the center of the screen. With keys 1 and 3, counterbalanced across participants, participants had to indicate whether the presented stimulus belonged to one or the other category. Importantly, participants were not offered more specific information, nor a category label, and were informed to guess in the first trials and use the feedback to derive the underlying category rule. Feedback was given immediately after the participants’ response, by coloring the fixation cross in *red* or *green* for 500 ms in case of a *wrong* or *right* response, respectively. To proceed to the next training block, participants were required to reach at least 50% accuracy after the first block of the training. Participants who did not reach this criterion had to repeat the first block^3^.

### EEG data analysis

#### Preprocessing

All EEG processing steps were carried out using Letswave 6 and 7 (http://nocions.webnode.com/letswave) in Matlab R2018a (The Mathworks, Inc.). EEG data was segmented in 76-s segments (2 s before and 4 s after each sequence), bandpass filtered (0.1 to 100 Hz) using a fourth-order Butterworth filter, and down-sampled to 256 Hz. For one participant who blinked excessively (more than two standard deviations above the sample mean, *M*(± SD) = 0.15 ± 0.16 times/s across all EEG trials), blinks were corrected by means of independent component analysis (ICA) using the *runica* algorithm [42] as implemented in EEGLAB. For this one participant, the component accounting for most of the variance and representing vertical eye movements was removed. Next, noisy electrodes were linearly interpolated from the three spatially nearest electrodes for eleven participants (for 10 out of 11 one electrode and for one participant two electrodes), outside of the proposed region of interest. All data segments were re-referenced to a common average reference. Finally, data segments were further cropped to contain an integer number of 1.2 Hz cycles (oddball frequency) into the sweep steps of 10 s (12 cycles, 2349 time bins in total).

#### Frequency-domain processing

The resulting segments were averaged for each stimulus dimension and each sweep step separately (i.e., separately for the 7 steps of the different sweep trials) and transformed into the frequency domain using a fast Fourier transform (FFT). The amplitude spectrum was computed with a spectral resolution of 0.1 Hz (1/10 s). The recorded EEG contains signals at frequencies that are integer multiples (harmonics) of the frequency at which images are presented (base stimulation frequency: 6 Hz). Crucially, only if the oddball stimuli are perceived as different of the base stimuli, significant EEG responses will also be present at the oddball frequency (1.2 Hz and harmonics). Since the EEG response at (harmonics of) these frequencies reflects both the overall noise level and the signal unique to the stimulus presentation, we used a baseline-corrected approach to describe the response in relation to the noise level [43, 44]. In particular, 12 surrounding frequency bins (eight bins on each side, excluding the two bins directly adjacent and the two bins with the most extreme value) were used to compute the baseline-corrected amplitude. Afterwards, for each sweep step, and stimulus dimension separately, we quantified the response by summing these baseline-corrected amplitudes across all consecutive significant harmonics and by regions of interest (ROI).

#### Determination of harmonics

We determined the harmonics for which the amplitude was significantly above noise using a *z*-score approach [43, 44]. For all segments, FFT amplitude spectra were averaged across subjects, then pooled across all electrodes and across electrodes in the relevant ROIs, and the resulting FFTs were then transformed in *z*-scores (computed as the difference between the amplitude at each frequency bin and the mean amplitude of the corresponding bins divided by the SD of amplitudes in these surrounding bins). Significant harmonics corresponded with a *z*-score above 1.64 (or *p* < 0.05, one-tailed). We focused on the EEG segments for which we expected the highest oddball activity, i.e., step 7 contrasting the two original endpoint stimuli of the assigned stimulus dimension in the FT-EEG sweep paradigm. Based on this criterion, we quantified oddball responses by summing five harmonics: harmonics 1 (1.2 Hz) to 6 (7.2 Hz) excluding the harmonic corresponding to the base stimulation frequency (6 Hz). In addition, the general visual response was quantified as the sum of the response at the base rate (6 Hz) and three consecutive harmonics (12 Hz, 18 Hz and 24 Hz).

#### Determination of ROIs

As in Vos et al., 2023 [45], we wanted to objectively select the regions of interest (ROIs) based on the data of all subjects. We determined the ROIs separately for the base (6 Hz) and oddball frequency (1.2 Hz) as we expected different patterns of activation for the different frequencies. Hence, we calculated the baseline-subtracted amplitude across all subjects, all stimuli and each electrode, and summed these across the significant base rate and oddball harmonics. All electrodes for which the baseline-subtracted amplitude of the response was significantly higher than the mean response (Bonferroni corrected) were retained and grouped in an ROI based on their location on the scalp. Similar to multiple studies assessing face categorization via FT-EEG [44, 46–49], the analysis of the general visual (base rate) response focused on a medial occipital ROI (MO: Oz, Iz, O1, O2), a region that has been found to be most responsive for base rate stimulation [43, 50]. The oddball analysis focused on a left (LOT: P9, PO7) and right occipito-temporal ROI (ROT: P10, P8, PO8).

### Statistical data analysis

Linear Mixed Models (LMMs) were tested using the package *afex* in R (https://www.R-project.org/; version 4.2.1) [51]. To assess discrimination sensitivity along the stimulus dimension (before and after category learning), an LMM was used separately for the baseline-subtracted oddball amplitude and behavioral d-prime with assessment moment (before and after category training) and either respectively sweep step (steps 2,4,6) or comparison (within- versus between- category) as fixed factors. To assess categorization performance, an LMM was used separately for accuracy, RTs, fitted threshold, and fitted slope as dependent measures with block as fixed factor. In each LMM, other fixed factors consisted of stimulus dimension (CR and AR) and group (ASC and NT) and a random intercept per participant was included to account for repeated testing. Each LMM was conducted on individual data per participant, aggregated across the three sweep trials or five presentations of each pair to assess neural or behavioral discrimination sensitivity (respectively) and different trials (15 trials per trial bin) or stimuli (15 responses per stimulus bin) to assess categorization performance during training. The dependent measures and additional factors will be clarified further in each subsection.

Post-hoc contrasts were performed on significant interaction effects with factor group (ASC and NT) and were tested with multiple comparison correction (Tukey method). Extremely outlying data-points (i.e., values above Q_3_ + 3xIQR or below Q_1_ – 3xIQR, in which Q_1_ and Q_3_ are the first and third quartile and IQR is the interquartile range (IQR = Q_3_ - Q_1_)) were removed. All assumptions in terms of linearity, normality and constant variance of residuals were verified and met for all LMMs. For correlation with participant characteristics (which were normally distributed), we used Pearson correlation. A Pearson’s *r* of 0.10 is considered as a small effect, 0.30 as a medium effect and 0.50 as a large effect. Effect sizes were reported as Cohen’s *d* for *t*-tests (i.e., *d* = 0.01: very small, *d* = 0.20: small, *d* = 0.50: medium, *d* = 0.80: large, *d* > 1.20: very large effect sizes) [52, 53] and as η^2^ for *F*-tests (i.e., η^2^ = 0.01: small, η^2^ = 0.06: medium, η^2^ = 0.14: large effect sizes) [54]. Effect sizes were reported with their 95% confidence interval (CI). Calculation of power and effect size, the Bayesian statistical analysis, as well as statistical results when excluding ASC participants with comorbidities, can be found in Supplementary Material.

#### Sweep FT-EEG measures

Orthogonal task performance and base activity (See Supplementary Material Figure S1)

***Oddball activity*** To compare neural discrimination sensitivity along the stimulus dimension, we specifically looked at the baseline-subtracted oddball amplitude of FT-EEG sweep step 2 and 6 to assess neural sensitivity for within the category and step 4 for across the trained category boundary. Sweep steps of interest (steps 2,4,6), stimulus dimension (CR or AR), ROI (left and right OT), assessment moment (before and after category training), and group (ASC and NT) were included as fixed factors. Difference in the direction of the sweep was not considered.

#### Behavioral measures

***Same-different discrimination task*** For the 2-AFC discrimination task, to specifically zoom in on learning effects and attention throughout the task, we first investigated accuracy and RTs across the different blocks (pre-determined: 4 blocks of 50 trials for each assessment). We applied an LMM to the overall accuracy and RTs with blocks (4 blocks), assessment moment (before and after category training) and group (ASC and NT) as fixed factors. We, indeed, found learning effects throughout the task mainly for the pre-assessment (see Supplementary Material Figure S2). Based on the obtained learning effect, for further analyses, blocks was added as an extra factor.

Our main analysis focused on the d-prime per specific pair type (i.e., comparison: within- versus between-category) to investigate the presence or absence of categorical perception. The d-prime was calculated as a bias-free index of accuracy for each discrimination pair (with hits corresponding with the percentage of different responses for the different pairs, and false alarms corresponding with the percentage of different responses for the same pairs [55]) per participant. Afterwards, an LMM was tested for d-prime with comparison (within- versus between-category), stimulus dimension (CR or AR), assessment moment (before and after category training), blocks (4 blocks), and group (ASC and NT) included as fixed factors. The blocks were included as a nested factor within assessment.

***Categorization training task*** For the 2-AFC categorization training, we averaged the accuracy responses of 150 trials (per block) to 10 trials bins (per block) to investigate category learning across time. Each trial bin thus consisted out of 15 averaged trials. We tested an LMM for accuracy and RT with stimulus dimension (CR or AR), trial bin (10 trial bins), block (3 blocks) and group (ASC and NT) included as fixed factors. The trial bins were included as a nested factor in the blocks.

For fitting of the category responses, we averaged the responses across the 50 stimuli to 10 stimulus bins of each 15 responses. In this way, we could fit the participants’ responses with a sampling of 15 responses per stimulus bin^4^. The percentage of the perceived category across the 10 stimulus bins was fitted via a psychometric curve to derive an individual threshold (i.e., category boundary) and slope of this category boundary for each participant (95% confidence interval) using the logistic function (see the *quickpsy* package in R [56]). For both these parameters an LMM was applied with stimulus dimension (CR or AR), block (3 blocks) and group (ASC and NT) included as fixed factors.

## Results

### More variability and inaccuracy during initial category learning in ASC compared to NT

#### ASC participants are less accurate at the initial stage of category learning

All participants successfully performed the categorization training. The average accuracy across the whole training was above 65% for each participant. Only one ASC participant had an average accuracy below 50% and only completed the first blocks of training (block 1 and 2). This participant was excluded for further analyses comparing discrimination sensitivity before and after category learning (see Results Sect. 2).

**Fig. 2:**
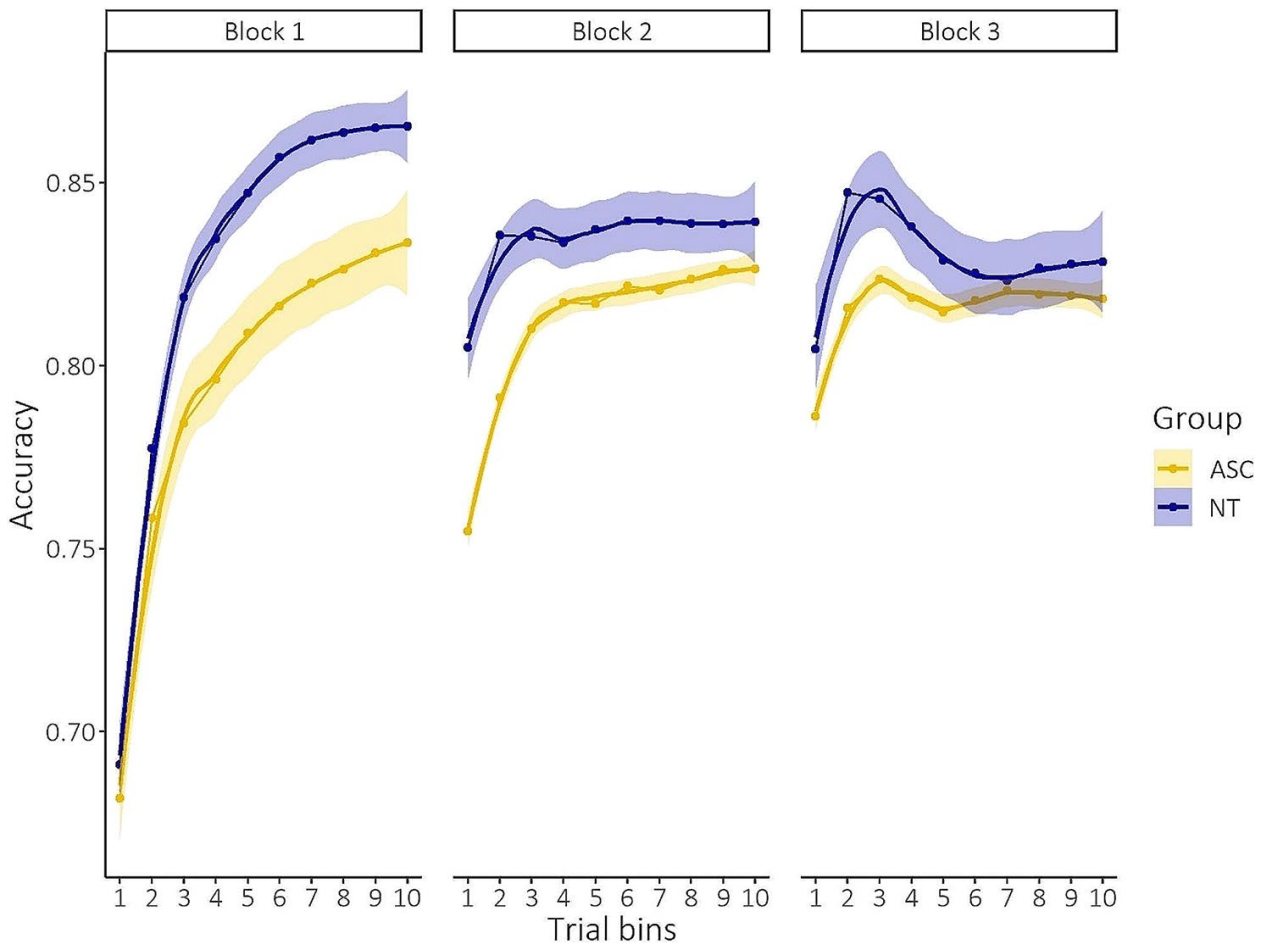
Results of participants’ accuracy during the category learning. Participants’ accuracy generally increased across each block. The accuracy was lower at block 2 and 3 (compared to block 1) because of an increased categorization difficulty of these blocks due to a higher stimulus sampling around the trained category boundary. ASC participants were slower in learning the categories. Differences between group (ASC and NT) were prominent at the initial block of category learning. *Mean values are plotted with 95% confidence interval

Figure 2 displays the results of participants’ accuracy during the category learning. We observed a significant main effect of trial bin (*F*(1,2156.93) = 141.22, *p* < 0.001, η^2^ = 0.06, CI: [0.05,1.00]), block (*F*(2,2157.05) = 52.30, *p* < 0.001, η^2^ = 0.05, CI: [0.03,1.00]) and group (*F*(1,96.94) = 4.43, *p* = 0.04, η^2^ = 0.04, CI: [0.00,1.00]). No significant main effect of stimulus dimension (*F*(1,96.94) = 2.71, *p* = 0.10) was present. In addition, we observed a significant interaction effect of trial bin x block (*F*(2,2156.93) = 57.96, *p* < 0.001, η^2^ = 0.05, CI: [0.04,1.00]), group x block x stimulus dimension (*F*(2,2157.05) = 3.28, *p* = 0.04, η^2^ = 0.003, CI: [0.00,1.00]) and a marginal significant effect of group x trial bin x block x stimulus dimension (*F*(2,2156.93) = 2.32, *p* = 0.098, η^2^ = 0.002, CI: [0.00,1.00]). These results point to a general lower accuracy for participants with ASC but also indicate that participants’ accuracy increased across the different trials and blocks, pointing to a learning effect during training. Post-hoc testing of the later interactions effects, specifically, revealed that differences between groups (ASC and NT) were specifically present at the initial block of category learning (*t*(87.9)_ASC−NT_=-2.13, *p* = 0.04, *d*=-0.46, CI: [-0.88,-0.03]) and for the CR stimulus dimension (*t*(88.3)_ASC−NT_=-2.02, *p* = 0.046, *d*=-0.43, CI: [-0.85,-0.01]). This indicates that ASC participants were slower in learning the categories, especially for the CR dimension. No other significant interaction effects were present (all *p* > 0.2).

For RTs, we found a significant main effect of trial bin (*F*(1,2139.76) = 124.67, *p* < 0.001, η^2^ = 0.06, CI: [0.04,1.00]) and block (*F*(2,2139.23) = 100.19, *p* < 0.001, η^2^ = 0.09, CI: [0.07,1.00]), in which RTs significantly decreased across trial bins and blocks for both groups. No main effect of group (*F*(1,82.60) = 0.51, *p* = 0.48) and stimulus dimension (*F*(1,82.60) = 0.22, *p* = 0.64) was found. We did find a significant interaction effect of trial bin x block (*F*(2,2139.30) = 88.20, *p* < 0.001, η^2^ = 0.08, CI: [0.06,1.00]), trial bin x stimulus dimension (*F*(2,2139.76) = 5.27 *p* = 0.02, η^2^ = 0.003, CI: [0.00,1.00]), group x block (*F*(2,2139.23) = 4.01 *p* = 0.02, η^2^ = 0.004, CI: [0.00,1.00]) and group x stimulus dimension (*F*(1,82.60) = 5.94 *p* = 0.02, η^2^ = 0.07, CI: [0.01,1.00]). Post-hoc testing showed that ASC participants responded significantly faster (RTs significantly decreased) after the first block (*t*(2141)_block1−2_=5.50, *p* < 0.0001, *d* = 0.24, CI: [0.15,0.32] and *t*(2140)_block1−3_=3.88, *p* = 0.0003, *d* = 0.17, CI: [0.08,0.25]) in line with their enhanced category learning after the initial block. In addition, post-hoc testing revealed that NT participants responded faster (lower RTs) for the CR dimension compared to ASC participants (*t*(72.0)_ASC−NT_ =1.81, *p* = 0.07, *d* = 0.42, CI: [-0.04,0.89]). This is in line with ASC participants’ difficulty for categorization in the CR dimension (cf. accuracy results). No other significant interaction effects were present (all *p* > 0.1).

#### ASC participants show more heterogeneity in the initial stage of category learning

**Fig. 3:**
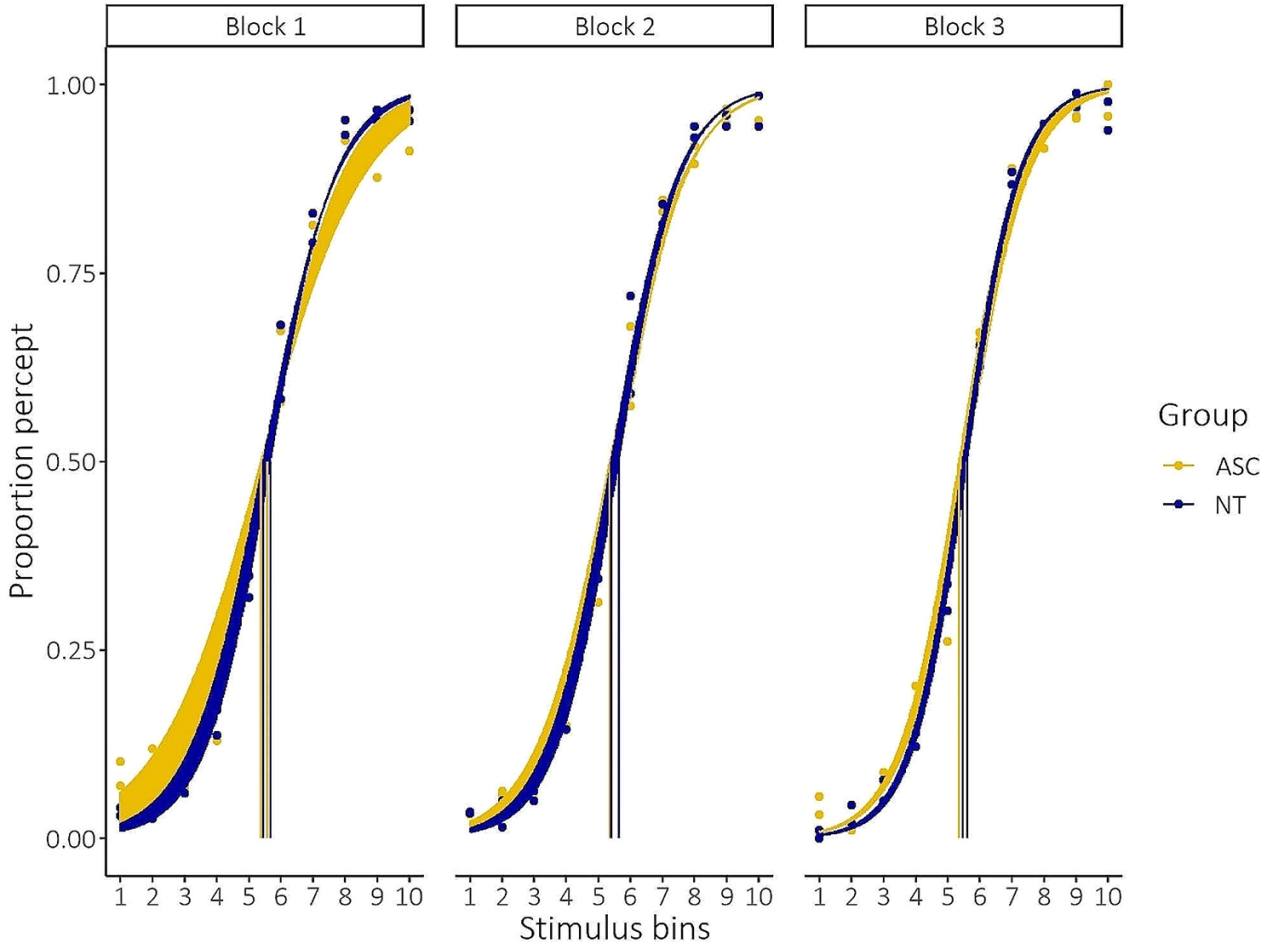
Results of participants’ fitted category boundary during category learning. The obtained category boundary (trained at midpoint of the stimulus dimension) in the first block did not significantly change in subsequent blocks. Participants did learn to be more precise in their assignment to the two different categories. Precision of category boundary was more variable across the different ASC participants, as evidenced by the larger variability in slope, especially in the initial block. *Mean values are plotted with participants’ individual curves

When fitting a logistic function to participants’ response (i.e., proportion of category ‘A’ or ‘B’ response) across the stimulus space, we can obtain two parameters for each participant and for each training block: the position of the category boundary (i.e., threshold) and the steepness of the category boundary (i.e., slope). Figure 3 displays the results of participants’ fitted category boundary during category learning. Pertaining to the position of the category boundary, we observed a significant main effect of stimulus dimension (*F*(1,69.73) = 14.08, *p* < 0.001, η^2^ = 0.17, CI: [0.05,1.00]). Post-hoc testing showed a slight bias in the location of the category boundary for the AR dimension (*t*(70.7)_AR−CR_=3.75, *p* = 0.0004, *d* = 0.89, CI: [0.40,1.38], see Supplementary Material Figure S5). No significant main effects of group (*F*(1,69.73) = 0.62, *p* = 0.43) or block were present (*F*(2,136.48) = 0.35, *p* = 0.7), nor any interaction effects (all *p* > 0.4). For the steepness of the category boundary, we observed a significant main effect of block (*F*(2,139.04) = 19.51, *p* < 0.001, η^2^ = 0.22, CI: [0.12,1.00]). No significant main effects of group (*F*(1,70.99) = 0.48, *p* = 0.49) or stimulus dimension were present (*F*(1,70.99) = 1, *p* = 0.32), nor any significant interaction effects (all *p* > 0.2). Bayesian statistical modelling confirmed these findings (Supplementary Material Figure S3). Using the Bayesian approach, we found an effect of block (estimate: 0.04, HDI: [0.03,0.05]) and level x block (estimate: 0.01, HDI: [0.00,0.01]).

However, when we tested the variability of obtained results, we could detect a higher variability in the slopes values of the ASC participants (*F*_*var*_(111, 110) = 0.63, *p* = 0.02). This effect was especially driven by the initial block (*F*_*var*_(37,37) = 0.31, *p* = 0.0005, see Fig. 3).

These results indicate that participants were able to quickly distinguish the shapes along the stimulus continua in two different categories. The Bayesian modeling approach points to a significant effect of block. The psychometric approach further specifies that the obtained category boundary did not significantly change in subsequent blocks, but that participants did learn to be more precise in their assignment to the two different categories (leading to a significant change in the steepness of the slope). We additionally observed significantly higher variability of the slope of the category boundary for the ASC participants, especially in the initial block. In conclusion, these results indicate that more participants with ASC are less precise/consistent in the initial phase of explicit category learning.

### Discrimination sensitivity only changes after explicit category learning in ASC compared to NT

#### Behavioral categorical perception is already induced by implicit learning in NT

**Fig. 4:**
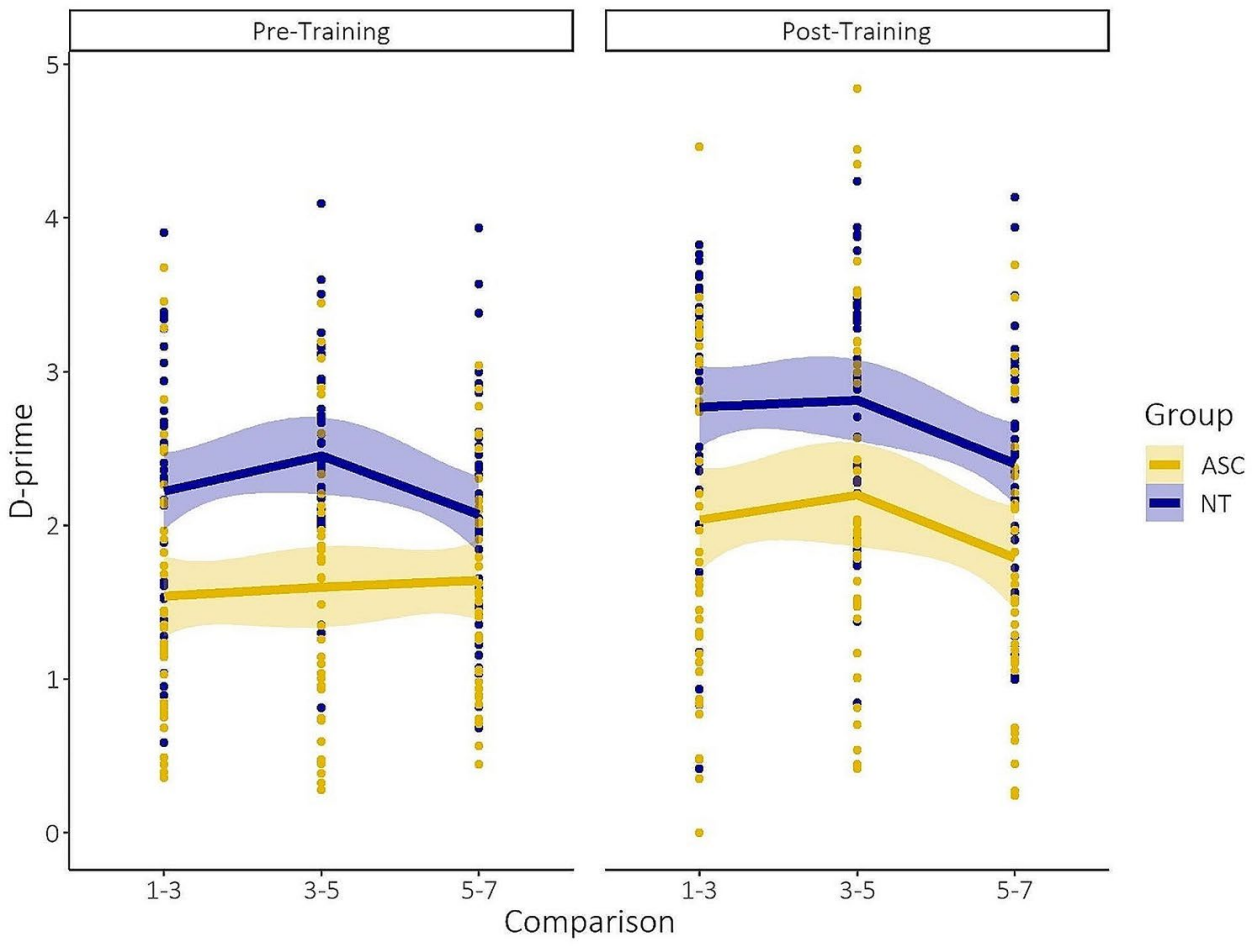
Results for the behavioral discrimination task. Stimulus pairs 1–3 and 5–7 reflect within category perceptual discrimination, while stimulus pair 3–5 reflects between category perceptual discrimination. **Left panel**: NT participants implicitly picked up the underlying categorical dimension (and therefore already showed a categorical perception effect) before training in comparison to ASC participants. **Right panel**: ASC participants only showed a categorical perception effect after training. Do (visually) note a systemic bias in discrimination sensitivity towards pair 1–3 (compared to pair 5–7). This bias seems to be mainly driven by the CR dimension (see Supplementary Material Figure S6). *Mean values (lines) are plotted with 95% confidence interval and participants’ individual values (dots)

Figure 4 displays the results for the behavioral discrimination task, both pre- and post-training. For d-prime, we observed a significant main effect of comparison (i.e., overall increased discrimination sensitivity for pair across the category boundary, *F*(1,1682.02) = 16.05, *p* < 0.001, η^2^ = 0.01, CI: [0.00, 1.00]), assessment moment (i.e., overall increased discrimination sensitivity after training; *F*(1,1682.02) = 77.07, *p* < 0.001, η^2^ = 0.04, CI: [0.03,1.00]), block (i.e., overall increased discrimination sensitivity across the different blocks, *F*(3,1682.02) = 24.81, *p* < 0.001, η^2^ = 0.04, CI: [0.03,1.00]), and group (i.e., overall lower discrimination sensitivity for ASC, *F*(1,73.75) = 19.37, *p* < 0.001, η^2^ = 0.21, CI: [0.09,1.00]). We did not observe a main significant effect of stimulus dimension (*F*(1,73.75) = 1.63, *p* = 0.21).

Most importantly, we observed a significant interaction effect of comparison x assessment moment x group (*F*(1,1682.02) = 3.93, *p* = 0.047, η^2^ = 0.002, CI: [0.00,1.00]). Post-hoc testing revealed that categorical perception (i.e., increased sensitivity for pairs that cross the category boundary compared to pairs within the category) is both present before (*t*(1682)_between−within_=3.02, *p* = 0.003, *d* = 0.15, CI: [0.05,0.24]) and after (*t*(1682)_between−within_=2.07, *p* = 0.04, *d* = 0.10, CI: [0.01,0.20]) categorization training in the NT group, while for the ASC group behavioral categorical perception is absent before training (*t*(1682)_between−within_=-0.05, *p* = 0.96, *d*=-0.003, CI: [-0.10,0.09]) and only present after explicit category learning (*t*(1682)_between−within_=2.97, *p* = 0.003, *d* = 0.14, CI: [0.05,0.24]). This implies that NT participants already implicitly pick up the underlying characteristics of the stimulus dimension, without explicit categorization training, and that this already influences their perception. ASC participants, on the other hand, are not able to implicitly incorporate the underlying dimensions of the stimuli, without explicit training (see Fig. 4, left panel). Consequently, their behavioral discrimination sensitivity is only influenced after explicit category training (see Fig. 4, right panel). Bayesian statistical modelling confirmed the main findings (Supplementary Material Figure S4). We found a clear interaction effect of comparison x assessment moment x group x stimulus dimension (estimate: 0.41, HDI: [0.04,0.78]). This interaction effect, again, indicates that NT participants implicitly picked up the underlying categorical dimension (and therefore already showed a categorical perception effect) before training in comparison to ASC who only showed a categorical perception effect after training. This effect seems to be mainly driven by the AR stimuli. This is in line with the observed marginal interaction effect of assessment moment x group x stimulus dimension (*F*(1,1682.02) = 2.86, *p* = 0.09, η^2^ = 0.002, CI: [0.00,1.00]) (see Supplementary Material Figure S6).

We also observed a significant interaction of block with assessment moment (*F*(3,1682.02) = 3.79, *p* = 0.01, η^2^ = 0.007, CI: [0.00,1.00]) and stimulus dimension (*F*(3,1682.02) = 3.73, *p* = 0.01, η^2^ = 0.007, CI: [0.00,1.00]) and its marginal interaction with comparison (*F*(3,1682.02) = 2.22, *p* = 0.08, η^2^ = 0.004, CI: [0.00,1.00]) and group (*F*(3,1682.02) = 2.23, *p* = 0.08, η^2^ = 0.004, CI: [0.00,1.00]). Most importantly, we observed a significant interaction between assessment moment x block x group (*F*(3,1682.02) = 3.25, *p* = 0.02, η^2^ = 0.006, CI: [0.00,1.00]). Post-hoc testing showed that the d-prime significantly increased across the consequent blocks, and this specifically for the NT participants in the pre-training assessment (*t*(1682)_block2−1_=4.41, *p* = 0.001, *d* = 0.20, CI: [0.10,0.30] and *t*(1682)_block3−2_=2.53, *p* = 0.03, *d* = 0.12, CI: [0.03,0.22]). This is in line with the significantly decreased RTs for the NT participants before training across the different blocks (Supplementary Material Figure S2) and could reflect the implicit learning of the NT participants. No other interaction effects were present (all *p* > 0.1).

#### Neural discrimination sensitivity changes only after explicit category learning in ASC

**Fig. 5:**
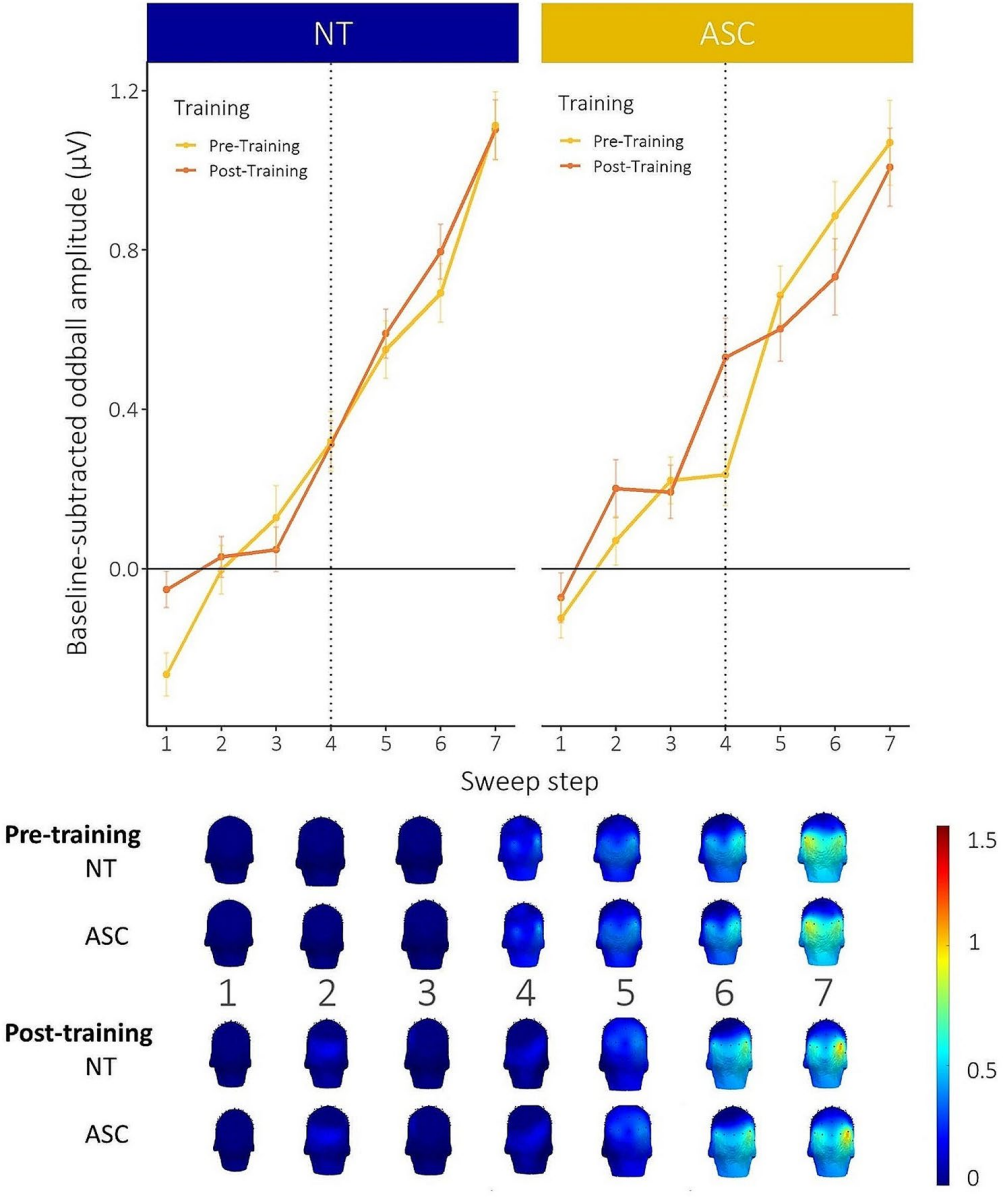
Oddball results for the neural FT-EEG sweep (averaged across both ROIs). Left panel: Analyses revealed that the neural sensitivity at the category boundary (see dashed line, i.e., hallmark of categorical perception) did not significantly differ after explicit category learning (compared to before) for NT participants. **Right panel**: We did find a significant increase in neural sensitivity at the category boundary after explicit category learning (compared to before) for the ASC participants. The bottom panel shows the head topographies along the 7 sweep steps. *Error bars correspond to standard errors of the mean

Figure 5 displays the results for the neural FT-EEG sweep oddball amplitudes. Using this direct neural approach, we observed a significant main effect of sweep step (*F*(2,741.11) = 128.98, *p* < 0.001, η^2^ = 0.26, CI: [0.21,1.00]), which is clearly represented in the increasing baseline-subtracted oddball amplitude along the dimension. We observed a marginal main effect of assessment moment (*F*(1,732.37) = 3.64, *p* = 0.06) and no effect of group (*F*(1,65.58) = 0.44, *p* = 0.51).

Most interestingly, we observed a significant three-way interaction of sweep step x assessment moment x group (*F*(2,732.39) = 5.08, *p* = 0.006, η^2^ = 0.01, CI: [0.00,1.00]). Post-hoc testing revealed that the neural sensitivity at the category boundary (i.e., hallmark of categorical perception) significantly increased after explicit category learning as compared to pre-training for ASC participants (*t*(737)_post−pre_=2.69, *p* = 0.007, *d* = 0.20, CI: [0.05,0.34]). This is not the case for the NT participants, who had similar levels pre- and post-training (*t*(736)_post−pre_=0.01, *p* = 0.99), which is in line with the behavioral discrimination data (in which NT already showed the categorical perception effect before the training).

Finally, we observed significant main effects of ROI (*F*(1,732.68) = 17.96, *p* < 0.001, η^2^ = 0.02, CI: [0.01,1.00]) and stimulus dimension (*F*(1,65.58) = 10.38, *p* = 0.002, η^2^ = 0.14, CI: [0.03,1.00]). This indicates a higher elicited baseline-subtracted oddball amplitude along the FT-EEG sweep for the right OT (*t*(737)_left−right_=-4.24, *p* < 0.001, *d*=-0.31, CI: [-0.46,-0.17]) and AR dimension (*t*(69.8)_AR−CR_=3.22, *p* = 0.002, *d* = 0.77, CI: [0.28,1.25]). We also observed a significant interaction effect of ROI x sweep step (*F*(2,732.67) = 3.86, *p* = 0.02, η^2^ = 0.01, CI: [0.00,1.00]), stimulus dimension x sweep step (*F*(2,741.11) = 8.46, *p* < 0.001, η^2^ = 0.02, CI: [0.01,1.00]), ROI x stimulus dimension (*F*(1,732.68) = 6.79, *p* = 0.009, η^2^ = 0.01), assessment moment x group x stimulus dimension (*F*(1,732.37) = 4.88, *p* = 0.03, η^2^ = 0.007, CI: [0.00,1.00]), sweep step x ROI x stimulus dimension (*F*(2,732.67) = 5.44, *p* = 0.005, η^2^ = 0.01, CI: [0.00,1.00]), and a marginal interaction effect of sweep step x group x stimulus dimension (*F*(2,741.11) = 2.90, *p* = 0.06, η^2^ = 0.008, CI: [0.00,1.00]). Post-hoc testing (on interaction effects with group) revealed that increased discrimination sensitivity after training was mainly due to the AR dimension for the ASC participants (*t*(737)_post−pre_=2.11, *p* = 0.03, *d* = 0.16, CI: [0.01,0.30]) and that ASC participants displayed a reduced neural category tuning for the CR dimension (i.e., an enhanced neural discrimination sensitivity towards the start of the CR dimension; *t*(737)_level2−4_=-1.21, *p* = 0.45, *d*=-0.09, CI: [-0.23,0.06], see Supplementary Material Figure S6). This is in line with behavioral results which showed increased category learning of ASC participants for the AR dimension during training (compared to the CR dimension) and a bias in behavioral discrimination sensitivity (for both groups) towards the start of the CR dimension (i.e., pair 1–3, see Fig. 4). No other interaction effects were present (all *p* > 0.1).

Finally, the lack of group differences in terms of orthogonal color change detection task performance and base-rate synchronization amplitude during FT-EEG assessments (Supplementary Material Figure S1) confirms that there are no systematic changes in processing demands (e.g., effort or attention) or brain synchronization and that potential differences in oddball activity between groups are thus due to effectively perceived stimulus differences.

### Correlations with participants’ characteristics

#### Learning differences are more pronounced across AQ characteristics

When we correlated accuracy of the three different categorization training blocks (averaged across the different trial bins) with questionnaire scores of the participants, we found a significant negative correlation with AQ characteristics for block 1 (*r*=-0.26, *p* = 0.02). This correlation indicates that participants with higher AQ scores performed worse in the categorization training. For the estimated threshold and slope for the fitted logistic function across the blocks, we only found a marginally significant negative correlation with AQ characteristics in block 1 for the slope values (*r*=-0.19, *p* = 0.09). This was mainly driven by the CR dimension (*r*=-0.30, *p* = 0.07). No significant correlations were found for the GSQ scores.

#### Differences in behavioral discrimination sensitivity are driven by AQ and GSQ characteristics

**Fig. 6:**
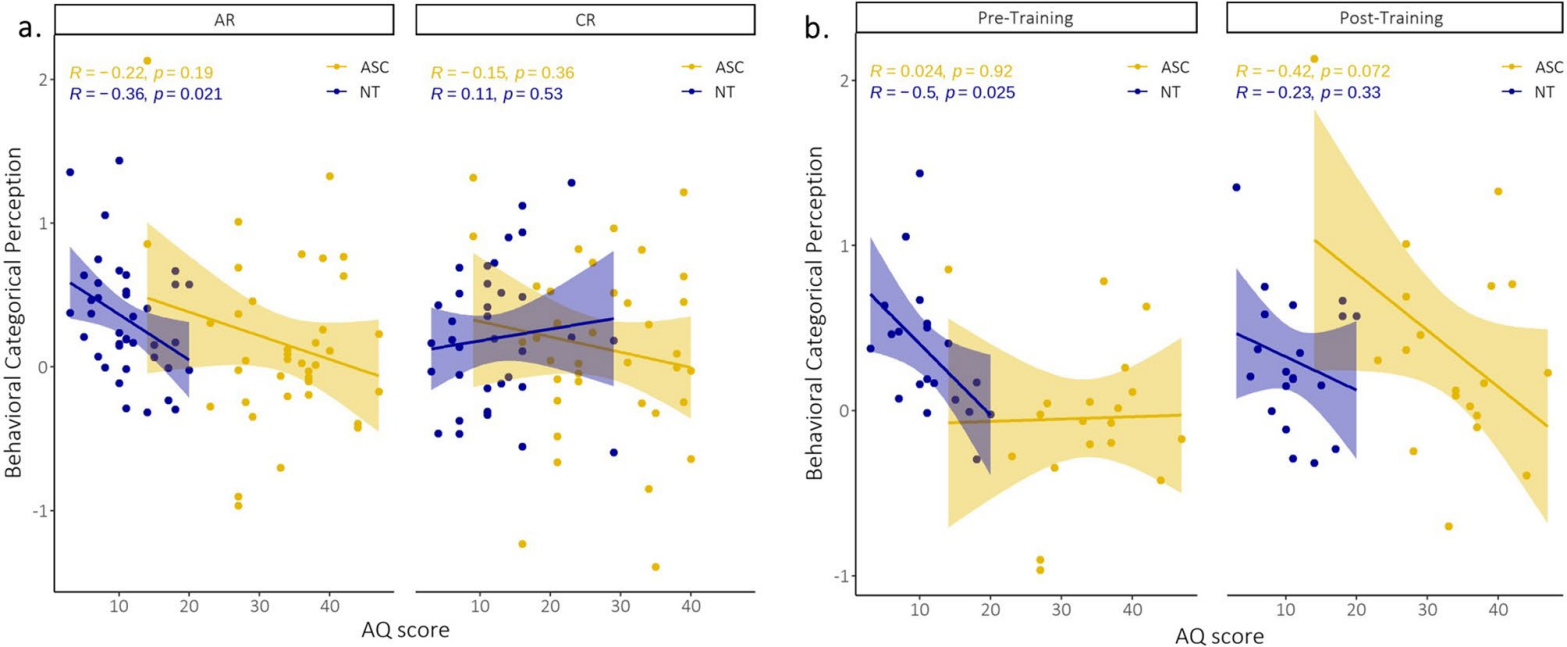
Correlations of behavioral categorical perception and participants characteristics. **(a)** We found a significant negative correlation of the behavioral categorical perception effect with AQ scores for the AR dimension. **(b)** This was specifically driven by the results before training for the NT participants. We also found a marginal significant negative correlation effect after training, specifically for the ASC participants

When we correlated the behavioral categorical perception effect (i.e., difference in discrimination sensitivity of pairs between versus within the category) with participants’ characteristics, we found a significant correlation with AQ scores for the pre-training assessment (*r*=-0.27, *p* = 0.02). This negative correlation seemed to be specifically driven by the AR dimension (*r*=-0.44, *p* = 0.005). This correlation indicates that participants with lower AQ scores are better able to implicitly pick up the underlying categorical dimension (and therefore already show a categorical perception effect for the dimension) before training in comparison to participants with higher AQ scores. This indicates that our main finding, that NT but not ASC participants already implicitly picked up the underlying dimensions of the stimuli without explicit training, could mainly be driven by their lower AQ characteristics. In Fig. 6, we can see that this effect is mainly driven by the NT participants (AR: *r*=-0.36, *p* = 0.02 in Fig. 6A and AR pre-training: *r*=-0.50, *p* = 0.03 in Fig. 6B). There was also a marginal correlation effect present post-training for the ASC participants (AR: *r*=-0.42, *p* = 0.07, Fig. 6B and both dimensions: *r*=-0.28, *p* = 0.09). This suggests that ASC participants with higher AQ traits showed a decreased categorical perception effect after training. We also found a significant negative correlation of the behavioral categorical perception effect with GSQ scores for the pre-training assessment (*r*=-0.29, *p* = 0.01). This was also mainly driven by the AR dimension (*r*=-0.37, *p* = 0.02).

When we examined the overall behavioral discrimination sensitivity in general (i.e., not taking into account the position along the category dimension and the pre- versus post-training assessment), we could find a significant negative correlation with both AQ- and GSQ-scores (AQ: *r*=-0.44, *p* < 0.0001 and GSQ: *r*=-0.26, *p* = 0.02). Moreover, for the GSQ scores this correlation was mainly driven by the hypo-sensitivity sub-score (*r*=-0.27, *p* = 0.02). This is in line with overall reduced d-prime values for the ASC participants and could suggest that this effect is mainly driven by their hypo-sensitivity characteristics.

Finally, when we correlated the neural categorical perception effect (i.e., sensitivity at the category boundary before and after training) and overall neural sensitivity with AQ-, and GSQ-scores across the participants, we found no significant correlation with participant characteristics.

## Discussion

In this study, we investigated categorization learning of highly controlled artificial (and thus unfamiliar) stimuli, as well as discrimination sensitivity of these stimuli before and after category learning in 38 ASC versus 38 NT adults. We combined direct and automatic FT-EEG measures with a standard psychophysical behavioral discrimination task, to obtain a full picture of possible differences in discrimination sensitivity and categorical perception before and after category learning.

### Autistic adults are slower and show more heterogeneity at the initial stages of category learning

Participants were assigned to be tested and trained on one of the two shape dimensions (aspect-ratio or curvature). Category learning consisted of an explicit training to assign a presented stimulus correctly to one of two (arbitrary) categories. Participants had to infer the category boundary along the underlying stimulus dimension and learn to group the stimuli in two categories based on feedback. No direct instructions were offered apart from the explicit feedback on every trial. We administered three training blocks with increasing difficulty level (i.e., by increasing the number of stimuli near the category boundary). We found a significant accuracy difference between the ASC and NT group, which was mainly driven by group differences in performance at the initial stage of training on the curvature dimension. Individual differences in training accuracy on the initial block were related to AQ scores. More specifically, reduced/slower learning at the beginning of the categorization task was associated with higher autistic traits.

While investigating the position and slope of the fitted category boundary, we found a general learning effect across the training (i.e., an increase in steepness of the category boundary), but no group difference. However, we did observe increased variability in the ASC group at the initial stage of training, suggesting that some autistic individuals show inconsistency at the initial stages of category learning. This was confirmed by a marginal negative correlation between the slope values and the AQ characteristics of the participants, indicating a less steep slope for higher autistic traits.

### Autistic adults only show a categorical perception effect after explicit category learning

When investigating behavioral discrimination sensitivity before and after category learning, we found an overall increased perceptual discrimination for the NT group. In addition, even prior to the training, the NT participants already showed the behavioral hallmark of categorical perception (i.e., increased discrimination sensitivity for pairs crossing the category boundary as compared to pairs falling within the same category), suggesting that NT participants were already able to implicitly derive the underlying stimulus dimension(s). Importantly, this was not the case for the autistic participants: before the training, their discrimination sensitivity was highly similar along the entire stimulus dimension, and only after explicit training did the categorical processing emerge and influence their percept. NT participants were mainly able to implicitly pick up the underlying dimension and consequently show the behavioral categorical perception effect for the aspect-ratio dimension compared to the curvature dimension. This aligns with the significant negative association between the behavioral categorical perception effect and AQ- and GSQ-characteristics which was specifically driven by AR dimension.

The neural FT-EEG data confirms that the automatic and direct neural oddball response can be used as neural discrimination sensitivity index (i.e., oddball response increases with increasing difference between the oddball and base stimulus). Even though we found no group difference in general neural discrimination sensitivity, we did observe a significant three-way interaction between sweep step, assessment moment, and group. This implicates an enhanced neural discrimination sensitivity at the category boundary after training (versus before) for the ASC group but not for the NT group. Similar to the behavioral data, this suggests that the NT participants already displayed maximal neural categorical processing before the training and that (in comparison) autistic participants’ automatic neural categorical processing improved significantly throughout the training. Moreover, the lack of group differences in terms of orthogonal color change detection task performance and base-rate synchronization amplitude during FT-EEG assessment(s) confirms that these observed group differences in baseline-subtracted oddball amplitude are due to perceived stimulus differences and not to systematic changes in processing demands (e.g., effort or attention) or brain synchronization.

### Difference in learning style in adults with autism

Overall, our results confirmed the findings of Soulières et al. (2007, 2011) in adolescents and young adults with autism [22, 23]. In particular, we replicated their results (on naturally categorically perceived figures [22]) and showed here that autistic individuals are less accurate and more autistic individuals are less consistent/precise at the initial stages of category learning on unfamiliar (i.e., artificial) stimuli. We also replicated that autistic adults do not show a spontaneous induced behavioral categorical perception effect when presented with unfamiliar (i.e., artificial) stimuli with pre-existing dimensions (i.e., elongation, curvature, etc.). However, after these autistic adults performed an explicit categorization training, we reported that these autistic individuals did reveal the typical behavioral categorical perception effect. This was also confirmed by our significant correlation with autistic characteristics and additional neural FT-EEG measurements, which show an increased neural discrimination sensitivity at the category boundary after category learning only for autistic adults.

In conclusion, autistic adults do not spontaneously detect underlying categorical dimensions (i.e., lack of categorical perception before training) and they have more difficulties learning to categorize (i.e., slower emergence during explicit training). This is in line with a difference in learning style as proposed by Qian & Lipkin (2011) [12]. As postulated by these authors, autistic individuals would be biased towards a look-up table style while NT individuals would be biased towards an interpolation style. This was later refined by Sapey-Triomphe, not as an inability to use interpolation rules, but as a disinclination to infer such rules in ASC [25]. Our results are in line with this view, showing that ASC individuals are not able to implicitly pick up a (INT-based) categorical dimension, but when they are explicitly trained (not via instructions but through feedback), they are able to infer the underlying (INT) category rule. We further refine this by showing that, even though they are able to explicitly learn, initially they are more inconsistent and less accurate in their training.

### Differences in processing of the different dimensions

We found differences in the processing of the curvature and aspect-ratio dimensions. We did not find any differences in the base amplitude of neural FT-EEG sensitivity assessment between the two dimensions. However, we found differences for the behavioral tasks and the oddball amplitude of neural FT-EEG sensitivity assessment. In general, the aspect-ratio stimuli elicited higher oddball amplitudes than the curvature dimension and the curvature dimension was more difficult to implicitly (before training by the NT) and explicitly (during training by the autistic individuals) pick up and/or use. Note that the category boundary for this curvature dimension is not positioned at an intrinsically evident position (because the dimension does not go from curved to fully straight), which also explain the bias in discrimination sensitivity for the CR dimension, specifically for the neural tuning in ASC participants. Hence, the reduced accuracy at the initial stage of category learning could be partly related to an initial inflexibility of the ASC individuals to adjust (as postulated by HIPPEA) [15, 57]. However, we could not find a significant effect in location of the category boundary for the CR dimension during training.

### Behavioral and neural discrimination sensitivity

In contrast with the prevailing idea of enhanced perceptual sensitivity and discrimination in ASC (e.g., Mottron et al. (2006) [6]), here, we show an overall reduced behavioral discrimination sensitivity of autistic versus NT participants. A possible explanation for the reduced behavioral discrimination in ASC could be the very short *simultaneous* presentation of the stimuli (i.e., 200 ms). This presentation duration is comparable to the *(sequential)* presentation time of stimuli during the FT-EEG sweep (i.e., 160 ms), where no group differences have been observed. However, due to the simultaneous presentation and the execution of the discrimination task (i.e., deciding whether the stimuli are same or different), behavioral discrimination requires more processing demands in both groups (and even more so in the ASC group). We could find a significant negative correlation of the overall behavioral discrimination sensitivity with autistic characteristics. This effect was mainly driven by their hypo-sensitivity characteristics. We could not find a significant correlation of neural discrimination sensitivity with autistic characteristics. This is similar to earlier results which failed to find correlations for neural sensory sensitivity and self-reported sensitivity and responsivity [58].

Finally, we note the right lateralization of neural perceptual discrimination response to the stimuli. We propose that this might be due to the asymmetry of these stimuli (i.e., the main body of the stimulus is positioned in the left visual field while the tips of the stimuli are positioned in the right visual field) which elicited a higher response in the corresponding right occipitotemporal cortex. This interpretation is in line with results on categorical processing of more naturally categorically perceived shapes [35] unlike faces which always elicited a right-lateralized response [44, 50]). Do note that the differences in size of the selected left and right ROI warrants a cautious interpretation.

### Limitations and future directions

Data-collection happened during the COVID-19 pandemic which led to dispersed data-collection between the different participants. Do note that the dispersed testing over time was similar for both groups (NT versus ASC). Although the large sample size enabled us to detect effects with rather small (approximate) effect sizes, we note that some of the obtained effect sizes for the interactions of interest were even smaller than the a priori-determined effect size. Moreover, 14 of the 38 ASC participants displayed a comorbid disorder. However, general trends and conclusions remained after excluding participants with comorbidities in the analyses (see Supplementary Material). In a follow-up study, we will use the full two-dimensional artificial space to specifically investigate category learning and subsequent generalization. For future studies, it would also be interesting to investigate categorization and perceptual discrimination of visual stimuli for which the dimensions underlying the construction of the shapes do not match the features and dimensions that people see in them, such as stimuli that consist of complex shapes defined by radial frequency components (RFCs) [59, 60]. Finally, it would be interesting to investigate these perceptual processes across development.

## Conclusions

In this study with 38 autistic versus 38 non-autistic adults, we investigated categorization learning of highly controlled artificial stimuli, as well as neural and behavioral discrimination sensitivity of these stimuli before and after categorization training. We found that autistic adults are less accurate and more uncertain at the initial stages of learning compared to non-autistic adults. In addition, whereas non-autistic participants implicitly grasp the category boundary of an artificial stimulus dimension, autistic individuals’ behavioral and neural discrimination sensitivity at the category boundary only changes after explicit category learning. These results replicate and extend earlier findings and provide evidence for a difference in sensory processing and learning in ASC.

### Electronic supplementary material

Below is the link to the electronic supplementary material.

**Supplementary Material 1:** Supplementary material

**Supplementary Material 2:** Movie ?CR_Sweep?

**Supplementary Material 3:** Movie ?AR_Sweep?

## Data Availability

Preprocessed EEG and behavioral data with analyses scripts necessary to reproduce the statistical analyses and figures in this manuscript will be made available at https://osf.io/n4krv/.

## Footnotes

1 To ensure that participants stay focused on the screen during this passive viewing, they will perform an orthogonal color change detection task (i.e., a task unrelated to the measure of interest in which participants typically have to detect color changes of the fixation cross during FT-EEG presentation).

2 The whole two-dimensional stimulus space will be used to assess generalization in a follow-up study.

3 Only one ASC participant had to repeat the first block (see Results Sect. 1).

4 A reduced number of stimuli were shown at the end-points of the stimulus dimension for block 2 and 3. Hence, the stimulus bins at the end of the dimension contained fewer trials than the stimulus bins around the center of the dimension.

## Abbreviations

ASC: Autism Spectrum Condition
DSM-5: Diagnostic and Statistical manual of Mental disorders
LUT: Look-up Table
INT: Interpolation
EEG: Electroencephalography
FT: Frequency-tagging
SNR: Signal-to-Noise Ratio
NT: Neurotypical
WAIS-IV-NL: Wechsler Adult Intelligence Scale IV-NL
AQ: Autism Spectrum Quotient
GSQ: Glasgow Sensory Sensitivity Scale
CMS: Common Mode Sense
DRL: Driven Right Leg
AR: Aspect-Ratio
CR: Curvature
2AFC: 2 Alternative Forced Choice Task
SD: Standard Deviation
ICA: Independent Component Analysis
FFT: Fast Fourier Transformation
ROI: Region of Interest
LOT and ROT: Left and Right Occipital Temporal Cortex
LMMs: Linear Mixed Models
Q: Quartile
IQR: Interquartile Range
RT: Reaction Times
CI: Confidence Interval
PSE: Point of Subjective Equality
HDI: Highest (Posterior) Density Interval
