# Supplementary material for: Discrimination sensitivity of visual shapes sharpens in autistic adults but only after explicit category learning
Source: Mol Autism. 2024 Jun 3;15:23. doi: 10.1186/s13229-024-00604-6 (PMC11149306; doi:10.1186/s13229-024-00604-6)
Supplement: Supplementary file 1 — Supplementary Material 1: Supplementary material [file 13229_2024_604_MOESM1_ESM.docx]

# Supplementary material

## Matching of participants for the stimulus dimensions separately

| **AR** | **NT** | **ASC** | ***p* (NT vs. ASC)** |
| --- | --- | --- | --- |
| Number of participants | 20 | 19 | - |
| Male / Female number | 11/9 | 11/8 | ns (*p*=0.94) |
| Age (years) | 29.55 (±8.62) | 33.30 (±9.25) | ns (*p*=0.20) |
| Left / Right-handed | 4/16 | 4/15 | ns (*p*=0.91) |
| Intelligence Quotient (FSIQ) | 111.5 (±14.87) | 108.79 (±15.80) | ns (*p*=0.58) |
| **CR** | **NT** | **ASC** | ***p* (NT vs. ASC)** |
| Number of participants | 18 | 19 | - |
| Male / Female number | 9/9 | 10/9 | ns (*p*=0.70) |
| Age (years) | 28.34 (±7.70) | 31.93 (±9.57) | ns (*p*=0.22) |
| Left / Right-handed | 1/17 | 3/16 | ns (*p*=0.41) |
| Intelligence Quotient (FSIQ) | 113.39 (±11.61) | 105.82 (±17.26) | ns (*p*=0.13) |
|  | ***p* (AR vs. CR)** | ***p* (AR vs. CR)** |  |
| Number of participants | - | - |  |
| Male / Female number | ns (*p*=0.43) | ns (*p*=0.75) |  |
| Age (years) | ns (*p*=0.65) | ns (*p*=0.64) |  |
| Left / Right-handed | ns (*p*=0.37) | ns (*p*=0.75) |  |
| Intelligence Quotient (FSIQ) | ns (*p*=0.67) | ns (*p*=0.67) |  |

**Table S1. Participants’ demographics matched across the two stimulus dimensions.** Group means (± standard deviations). Results of t-tests and chi-square test: ns: non-significant (*p*>0.05).

## Calculation of power and effect size

We decided to primarily employ mixed models in our analyses, because mixed models are vastly superior in controlling for Type I errors than alternative approaches especially in case of repeated measures [1,2]. Unfortunately, due to the way in which variance is partitioned in linear mixed models [3], there is no consistently established way to calculate standard effect sizes for individual model terms such as main effects or interactions. It is, however, possible to get approximations of most of the effect size indices. We approximated η^2^ (which is equivalent to R^2^) by the ratio of Signal^2^/(Signal^2^+Noise^2^), with the “noise” having all other “signals” (from other fixed or random effects) partialed out. This conversion of the F-statistic is based on Friedman [4]. To compute the effect size of post-hoc contrasts, we computed Signal/Noise with the “signal” representing the difference between two means. This is akin to Cohen’s d, and is a close approximation when comparing two groups of equal size [5,6].

To calculate our sensitivity to detect differences (at a power of 80%), we used the program G*Power (version 3.1.9.7)[7]. This is again an approximation because this program can also not take into account random intercept of the mixed models. For the discrimination sensitivity measures, we have an approximate sensitivity to detect effect sizes (Cohen’s *f*) of 0.27 (η^2^=0.07, between-factors), 0.18 (η^2^=0.03, within-factors) and 0.18 (η^2^=0.03, within-between interaction). For the categorization training, we have an approximate sensitivity to detect effect sizes (Cohen’s *f*) of 0.28 (η^2^=0.07, between-factors), 0.13 (η^2^=0.02, within-factors), 0.13 (η^2^=0.02, within-between interactions).

## No differences in attention throughout the FT-EEG sweep experiment

### **No differences in performance on the orthogonal task during FT-EEG assessment between ASC and NT**

In case participants responded within 1 s after the onset of the fixation cross color change (during the orthogonal task), this answer was scored as correct and reaction time (RT) was included. We report the average accuracy and RT with standard error. An LMM with stimulus dimension (CR or AR), assessment moment (before and after category training), and group (ASC and NT) as fixed factors was tested for both accuracy and RT.

Participants successfully performed the orthogonal color change detection task during the FT-EEG sweep. Only one ASC participant had an average accuracy below 50%. This participant was excluded for further EEG analyses (see Results Sect. 2). After exclusion, both groups showed similar accuracies, with average accuracy M=0.97±0.01 and average RT *M*=0.40±0.01 for ASC participants and with average accuracy M=0.98±0.005 and average RT *M*=0.37±0.005 for NT participants.

For accuracy, no significant main effect of group (*F*(1,71.00)=1.32, *p*=0.26), assessment moment (*F*(1,71.00)=1.37, *p*=0.25), stimulus dimension (*F*(1,71.00)=0.30, *p*=0.59) nor any interaction effect between these factors was observed (all *p*>0.2). For RTs, significant main effects of group (*F*(1,71.00)=8.16, *p*=0.006, η^2^=0.10, CI: [0.02,1.00], with higher RTs for ASC participants *t*(71)_ASC-NT_=2.86, *p*=0.006, *d*=0.68, CI: [0.20,1.15]) and assessment moment (*F*(1,71.00)=12.78, *p*<0.001, η^2^=0.15, CI: [0.05,1.00] with higher RTs in the post-training assessment *t*(71)_post-pre_=3.58, *p*=0.0006, *d*=0.85, CI: [0.36,1.33]) were observed. No significant main effect of stimulus dimension (*F*(1,71.00)=0.11, *p*=0.74) nor any interaction effect was observed (all *p*>0.2).

The absence of any significant effect for accuracy suggests that processing demands were similar between the two groups (ASC and NT) and remained the same no matter which stimulus dimension was presented (AR or CR) and whether the participants were assessed before or after training. We do note significantly slower reaction times of ASC participants and of all participants after training (compared to before training), which could be due to overall slower responses of ASC participants and tiredness of all participants towards the end of the experiment, respectively.

### **No differences in general visual base responses between ASC and NT**

We tested an LMM on the summed baseline-corrected base amplitudes for the MO region. Sweep steps (7 steps), stimulus dimension (CR or AR), assessment moment (before and after category training), and group (ASC and NT) were included as fixed factors. Difference in the direction of the sweep was not considered. Base-rate brain synchronization responses for the MO ROI for the FT-EEG sweep experiment (before and after training) are displayed in Figure S1. Base activity was comparable between group (*F*(1,69.93)=2.36, *p*=0.13) and stimulus dimension (*F*(1,69.93)=0.11, *p*=0.74), but there was a significant main effect of assessment moment (*F*(1,873.93)=4.46, *p*=0.04, η^2^=0.006, CI: [0.00,1.00]) and sweep step (*F*(6,874.11)=42.34, *p*<0.001, η^2^=0.23, CI: [0.18,1.00]), with lower responses towards the later assessment moment and later sweep steps. No interaction effects were present (all *p*>0.1). This suggests that processing demands were similar between the two groups (ASC and NT) and remained the same no matter which stimulus dimension was presented (AR or CR). We did find lower values whether the participants were assessed after training (in line with slower reaction times on the orthogonal color change detection task), perhaps pointing to fatigue towards the end of the study, and significantly lower values along the FT-EEG sweep paradigm, pointing to a slight habituation effect.


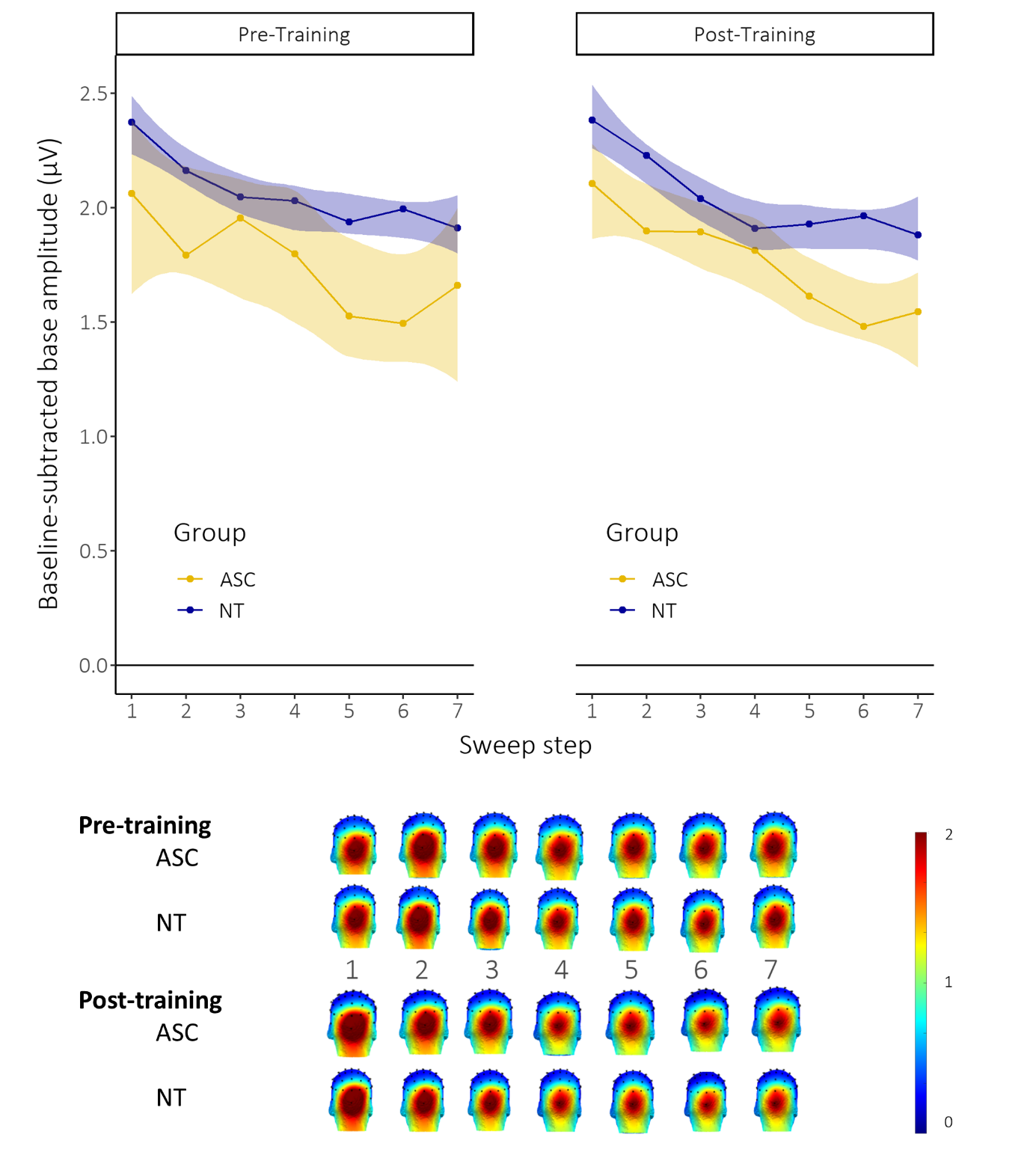


**Figure S1. Base-rate brain synchronization responses for the FT-EEG sweep experiment.** Base activity was comparable between groups. This suggests that processing demands were similar between the two groups (ASC and NT). We do note significant lower values after training, pointing to a fatigue-effect and lower values along the FT-EEG sweep paradigm, pointing to a slight habituation effect. The bottom panel shows the head topographies along the seven sweep steps. *Mean values are plotted with 95% confidence interval

## Assessing potential learning effect in the discrimination task (accuracy and RT across blocks)

For accuracy, we found a significant main effect of block (*F*(3,516.06)=18.85, *p*<0.001, η^2^=0.10, CI: [0.06, 1.00]), assessment moment (*F*(1,516.06)=70.72, *p*<0.001, η^2^=0.12, CI: [0.08,1.00]) and group (*F*(1,74.00)=22.27, *p*<0.001, η^2^=0.23, CI: [0.10, 1.00]). In addition, we found a significant interaction effect of block x assessment moment (*F*(3,516.06)=5.05, *p*=0.002, η^2^=0.03, CI: [0.01,1.00]), which points to the accuracy increasing significantly more during the discrimination task before training as compared to after training. This indicates a learning effect for both groups pre-training. No other interaction effects were present (*p*>0.1). For RTs, we also found a significant main effect of block (*F*(3,507.46)=45.32, *p*<0.001, η^2^=0.21, CI: [0.16,1.00]), assessment moment (*F*(1,507.34)=188.51, *p*<0.001, η^2^=0.27, CI: [0.22,1.00]) and an interaction effect of block x assessment moment (*F*(3,507.34)=3.92, *p*=0.009, η^2^=0.02, CI: [0.00,1.00]. This interaction effect with decreasing RTs across the different blocks before training is in line with the effect of increasing accuracy, again pointing to a learning effect pre-training. No main effect of group was present (*F*(1,73.25)=0.05, *p*=0.82). However, a marginal interaction effect of block x assessment moment x group (*F*(3,507.34)=2.18, *p*=0.09, η^2^=0.01, CI: [0.00,1.00]) revealed that the RTs for NT participants significantly decreased before training (as compared to after training) across the different blocks, which could specifically pinpoint to the implicit learning (of the underlying dimension) by the NT participants. No other interaction effects were present (*p*>0.1).


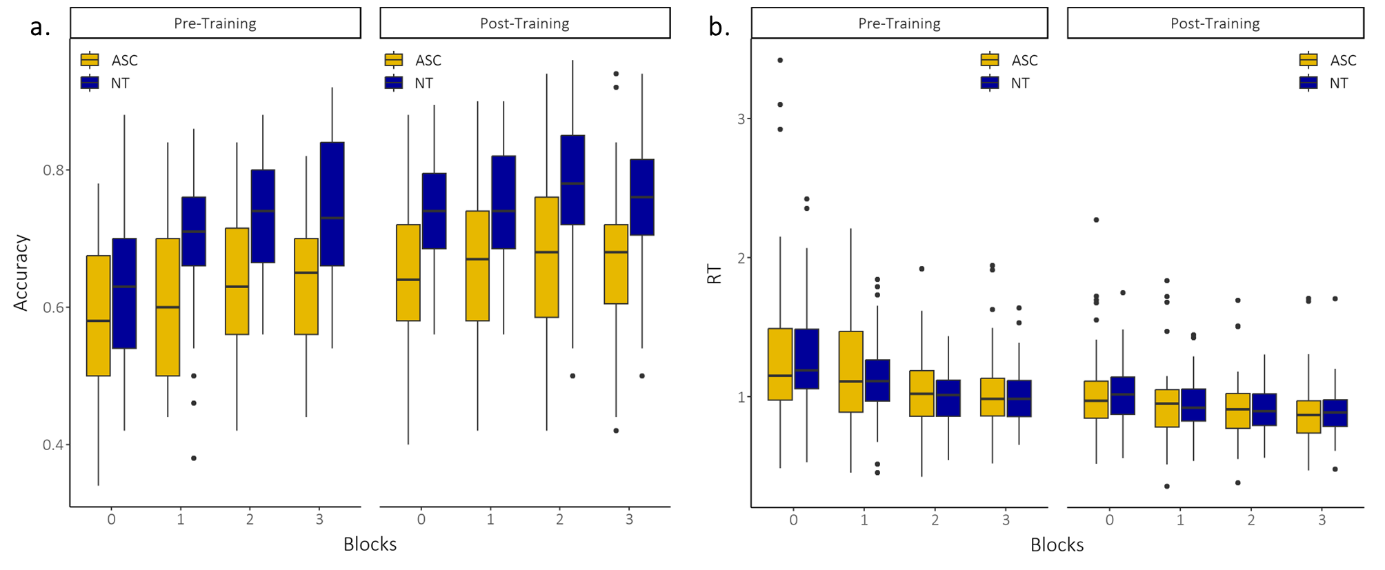


**Figure S2. Assessing potential learning effect in the discrimination task. (a)** Accuracy and **(b)** RT across blocks.

## The Bayesian approach for categorization and discrimination

LMMs were tested on calculated d-prime or psychometric fitted values as outcome variable (respectively for the discrimination task and categorization training). To get a more direct statistical assessment of performance on the discrimination task and categorization training, we used a Bayesian generalized linear mixed modelling framework with the obtained participants responses (i.e., same/different for the discrimination task and category A/B for the categorization training) as outcome variable. For the Bayesian approach we relied on the R package *brms* [8]. The categorical variables were always dummy-coded. For each model we ran four chains with 5,000 to 6,000 iterations each and the *adapt delta* parameter was set to 0.80. For each model, we checked that the model yielded MCMC chains that were converging, according to diagnostics such as effective sample size, the R-hat statistic, and the absence of divergent transitions. From the resulting model we can estimate the same parameters (as the frequentist approach) using the approach of Knoblauch and Maloney for the location of the point of subjective equality (PSE, which relates to the category boundary [9]) and other effect parameters (for d-prime). Effect estimates were reported with their 95% highest posterior density interval (HDI).

### **Bayesian approach for categorization training**

Using a Bayesian approach of the categorization training, a logit link function was used to model the sigmoidal pattern of the psychometric function on the stimulus levels. A Student-t prior was put on all regression weights, and an inverse-gamma prior on the parameters capturing the random effects. The fitted model was the following: 𝑝𝑒𝑟𝑐𝑒𝑝𝑡 ~ 1+ level ∗ stimulus dimension ∗ block ∗ group + (1+level|subject). The variable *percept* corresponds to the proportion of one percept and/or respective category group and variable *level* corresponds to the level along the stimulus dimension (indicative for the respective category). As outlined in Knoblauch and Maloney et al. (2012), the location, which corresponds to the 50% threshold or PSE, can be derived as follows: 𝑙𝑜𝑐𝑎𝑡𝑖𝑜𝑛=−(𝐼𝑛𝑡𝑒𝑟𝑐𝑒𝑝𝑡⁄𝑆𝑙𝑜𝑝𝑒)[9]. Next to the PSE, which indicates the perceived category boundary, we were also interested in the steepness of the slope at this category boundary, which indicates the precision of the learned category boundary. We found an effect of level (estimate: 0.04, HDI: [0.03,0.05], an interaction of level x block (estimate: 0.01, HDI: [0.00,0.01]). No other effects were found.


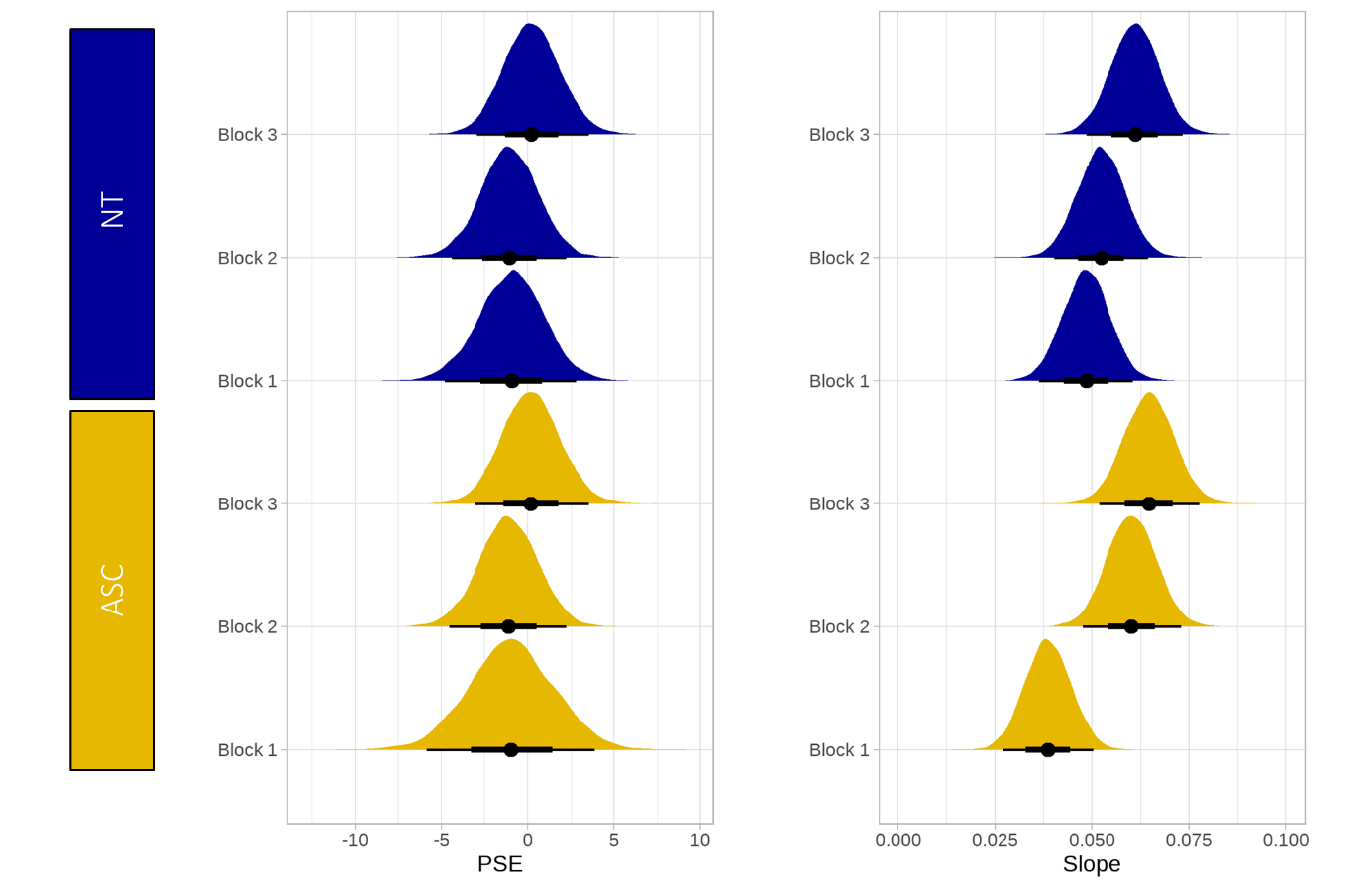


**Figure S3. Bayesian posterior distribution of PSE (left panel) and slope (right panel) values.**

### **Bayesian approach for discrimination task**

For the Bayesian approach of the discrimination task, a probit link function was used, and the fitted model was the following: 𝑟𝑒𝑠𝑝𝑜𝑛𝑠𝑒 ~ type ∗ comparison ∗ stimulus dimension ∗ assessment moment ∗ group + (type| subject). Here the variable *response* corresponds to the response given by the participant (same or different) and the variable *type* refers to whether the stimuli of the pair were the same or different. From this model we evaluated the response criterion c, which corresponds to -β0, and d prime, reflected in the other effect parameters. We found a main effect of type (i.e., same versus different pairs, estimate 0.90, HDI: [0.68,1.12]), of assessment moment (estimate: -0.16, HDI: [-0.27,-0.05]), an interaction effect of type x assessment moment (estimate: 0.46, HDI: [0.31,0.61]) and comparison x assessment moment (estimate: 0.20, HDI: [0.00,0.39]). More importantly, we found a clear interaction effect of comparison x assessment moment x group x stimulus dimension (estimate: 0.41, HDI: [0.04,0.78]). This interaction effect indicates that NT participants spontaneously picked up the underlying categorical dimension (and therefore already showed a categorical perception effect) before training in comparison to ASC who only showed a categorical perception effect after training. This effect seems to be mainly driven by the AR stimuli. This is confirmed by the interaction of comparison x assessment moment x stimulus dimension (estimate: -0.37, HDI: [-0.65,-0.09]). No other effects were found.


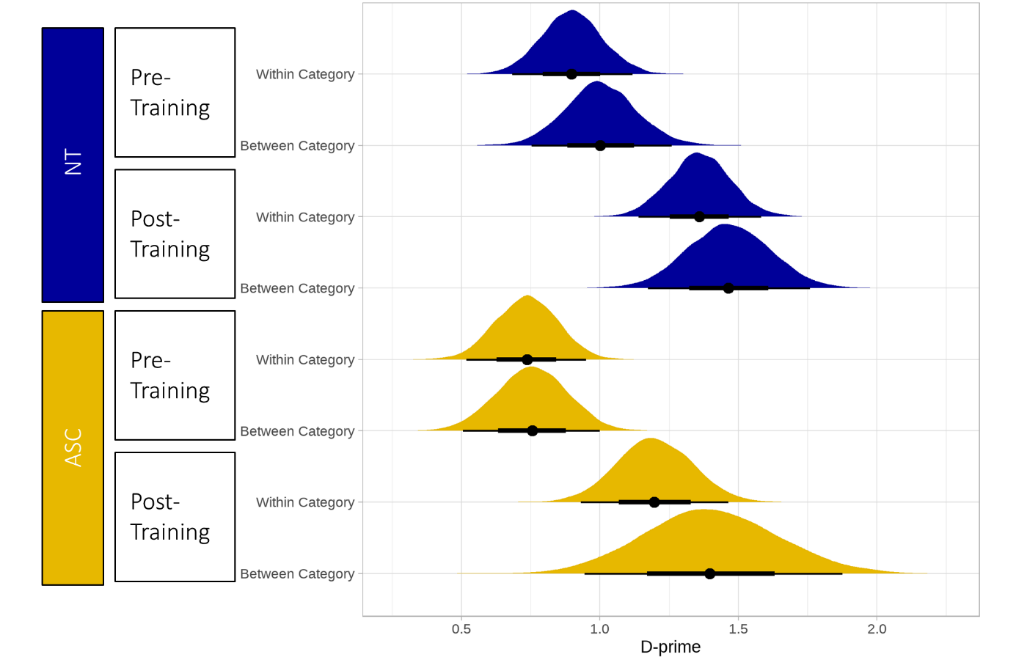


**Figure S4. Bayesian posterior distribution of D-prime values.**

## Results for the different stimulus dimensions separately


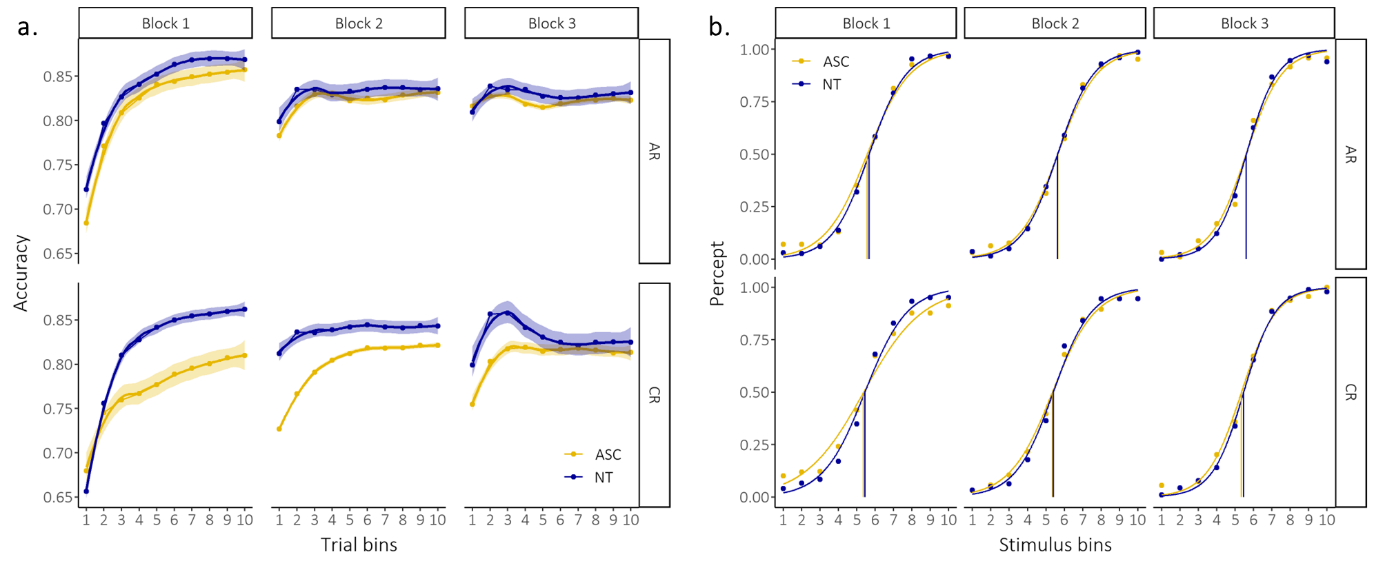


**Figure S5. Results during the category training.** **(a)** ASC participants were slower in learning the categories. Accuracy differences between group (ASC and NT) were specifically present at the initial block of category learning of the CR dimension. *Mean values are plotted with 95% confidence interval **(b)** The obtained category boundary in the first block did not significantly change in subsequent blocks. Participants did learn to be more precise in their assignment to the two different categories. Precision of category boundary was more variable across the different ASC participants, especially in the initial first block. *Mean values are plotted with indication of threshold


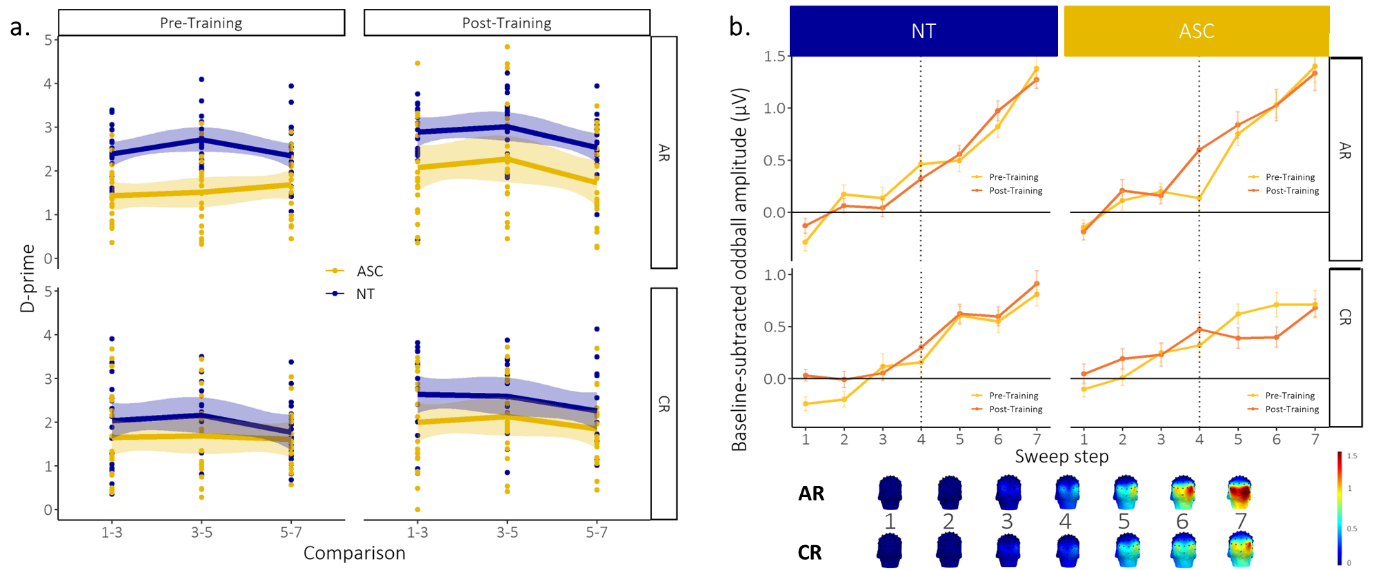


**Figure S6. Results for the discrimination sensitivity.** **(a) Behavioral discrimination sensitivity. Left panel:** NT participants implicitly pick up the underlying AR dimension (and therefore already show a categorical perception effect) before training in comparison to ASC participants. **Right panel:** ASC participants only show a categorical perception effect after training. *Mean values (lines) are plotted with 95% confidence interval and participants’ individual values (dots) **(b) Neural discrimination sensitivity. Left panel:** Analyses revealed that the neural sensitivity at the category boundary (see dashed line, i.e., hallmark of categorical perception) did not significantly differ after explicit category learning (compared to before) for NT participants. **Right panel:** We did find a significant increase in neural sensitivity at the category boundary after explicit category learning (compared to before) for the ASC participants, especially in the AR dimension. *Error bars correspond to standard errors of the mean

Results excluding participants with comorbidities

The participants with ASC but excluding the ones with comorbidities (i.e., resulting in 24 participants with ASC and no comorbidities) were still matched with NT on gender (*p*=0.77), age (*p*=0.11), laterality (*p*=0.61), and intelligence quotient (*p*=0.18) across both stimulus dimensions together^[[1]](#footnote-1)^. Given this, we repeated the statistical analysis for these participants without stimulus dimension as a fixed factor.

For the accuracy during categorization training, we found a significant main effect of trial bin (*F*(1,1781.93)= 100.39, *p*<0.001, η^2^=0.06, CI: [0.04,1.00]) and block (*F*(2,1781.92)=63.40, *p*<0.001, η^2^=0.07, CI: [0.05,1.00]). We also found a marginal main effect of group (*F*(1,95.02)=3.07, *p*=0.08, η^2^=0.03, CI: [0.00,1.00]). In addition, the LMM revealed a significant interaction of trial bin x block (*F*(2,1781.94)=52.91, *p*<0.001, η^2^=0.06, CI: [0.04,1.00]). The marginal main effect of group replicates the difference in accuracy during categorization training between both groups. Additionally, we observed a significant main effect of group for the threshold values during the categorization training (*F*(1,57.29)=5.80, *p*=0.02, η^2^=0.09, CI: [0.01,1.00]), which was not present in the original analysis. These threshold values showed a significantly higher variance in the autism group (*F*(110,67)=0.50, *p*=0.001, η^2^=0.09, CI: [0.01,1.00]). We also replicated the significant difference in variance for the slope values when comparing ASC to NT (across the whole training: *F*(111,68)=0.61, *p*=0.02 and in the first block: *F*(37,23)=0.29, *p*=0.0009). The correlation of AQ scores with accuracy and slope values for the first block during training remained significant (accuracy: *r*=-0.32, *p*=0.01; slope: *r*=-0.26, *p*=0.04).

For the behavioral discrimination sensitivity, we observed a significant main effect of comparison (*F*(1,1396)=18.69, *p*<0.001, η^2^=0.01, CI: [0.01,1.00]), assessment moment (*F*(1,1396)=68.47, *p*<0.001, η^2^=0.05, CI: [0.03,1.00]), block (*F*(3,1396)=19.62, *p*<0.001, η^2^=0.04, CI: [0.02,1.00]), and group *F*(1,61.49)=12.83, *p*<0.001, η^2^=0.17, CI: [0.05,1.00]). Additionally, we observed significant interaction effect of block with group (marginally significant; *F*(3,1396)=2.55, *p*=0.05, η^2^=0.005, CI: [0.00,1.00]) and assessment moment (*F*(3,1396)=3.72, *p*=0.01, η^2^=0.008, CI: [0.00,1.00]). Most importantly, we observed the following significant interaction effects with group: comparison x assessment moment x group (*F*(1,1396)=4.06, *p*=0.04, η^2^=0.003, CI: [0.00,1.00]) and assessment moment x block x group (marginally significant; *F*(3,1396)=2.10, *p*=0.098, η^2^=0.005, CI: [0.00,1.00]). Post-hoc testing revealed that categorical perception (i.e., increased discrimination sensitivity across the category boundary compared to within the category) was present in the NT group before and after training (pre-training: *t*(1396)=3.04, *p*=0.002, *d*=0.16, CI: [0.06, 0.27]; post-training: *t*(1396)=2.25, *p*=0.02, *d*=0.12, CI: [0.02,0.23]), while discrimination sensitivity only showed categorical tuning after training in the ASC group (pre-training: *t*(1396)=0.30, *p*=0.77, *d*=0.02, CI: [-0.09,0.12]; post-training: *t*(1396)=3.31, *p*=0.001, *d*=0.18, CI: [0.07, 0.28]). Additionally, only NT participants showed a significant implicit learning effect pre-training (*t*(1396)_block2-1_=4.10, *p*=0.0001, *d*=0.22, CI: [0.11,0.32]; *t*(1396)_block3-2_=2.51, *p*=0.03, *d*=0.13, CI: [0.03,0.24]). These trends are in line with the effects found in the total ASC versus NT group. We also replicated the correlation of the behavioral categorical perception effect with AQ scores (NT for AR dimension: *r*=-0.36, *p*=0.02 and for AR pre-training: *r*=-0.50, *p*=0.03; ASC for CR dimension: *r*=-0.50, *p*=0.02 and post-training, across both dimensions: *r*=-0.36, *p*=0.09) and GSQ scores (pre-training: *r*=-0.32, *p*=0.01 and AR pre-training: *r*=-0.33, *p*=0.06).

For the neural discrimination sensitivity, we observed a significant main effect of sweep step (*F*(2,636.02)=97.70, *p*<0.001, η^2^=0.24, CI: [0.19,1.00]), assessment moment (*F*(1,625.29)=4.00, *p*=0.046, η^2^=0.006, CI: [0.00,1.00]), and ROI (*F*(1,624.81)=14.11, *p*<0.001, η^2^=0.02, CI: [0.01,1.00]). We, additionally, observed a significant interaction effect of sweep step x ROI (*F*(2,624.81)=3.47, *p*=0.03 η^2^=0.01, CI: [0.00,1.00]). Most importantly, we observed the following significant interaction effect of sweep step x assessment moment x group (*F*(2,625.29)=3.24, *p*=0.04 η^2^=0.01, CI: [0.00,1.00]). Post-hoc testing revealed that significant differences in oddball activity (i.e., neural discrimination sensitivity) at the trained category boundary when comparing pre-and post assessment were only present in the ASC group (ASC: *t*(629)_post-pre_=2.17, *p*=0.03, *d*=0.17, CI: [0.02,0.33]; NT: *t*(626)_post-pre_=-0.07, *p*=0.95, *d*=-0.005, CI: [-0.16, 0.15]). This is a similar effect as in our original analysis (using the total amount of participants). Again, no significant correlations with AQ and GSQ scores were present.

## References

1. Barr DJ, Levy R, Scheepers C, Tily HJ. Random effects structure for confirmatory hypothesis testing: Keep it maximal. J Mem Lang. 2013 Apr;68(3):255–78.

2. Judd CM, Westfall J, Kenny DA. Treating stimuli as a random factor in social psychology: A new and comprehensive solution to a pervasive but largely ignored problem. J Pers Soc Psychol. 2012;103(1):54–69.

3. Rights JD, Sterba SK. Quantifying explained variance in multilevel models: An integrative framework for defining R-squared measures. Psychol Methods. 2019 Jun;24(3):309–38.

4. Friedman H. Simplified Determinations of Statistical Power, Magnitude of Effect and Research Sample Sizes. Educ Psychol Meas. 1982 Jun 7;42(2):521–6.

5. Wolf F. Meta-Analysis. 2455 Teller Road, Newbury Park California 91320 United States of America: SAGE Publications, Inc.; 1986.

6. Rosnow RL, Rosenthal R, Rubin DB. Contrasts and Correlations in Effect-Size Estimation. Psychol Sci. 2000 Nov 6;11(6):446–53.

7. Faul F, Erdfelder E, Lang AG, Buchner A. G*Power 3: A flexible statistical power analysis program for the social, behavioral, and biomedical sciences. Behav Res Methods. 2007 May;39(2):175–91.

8. Bürkner PC. brms : An R Package for Bayesian Multilevel Models Using Stan. J Stat Softw. 2017;80(1).

9. Knoblauch K, Maloney LT. Modeling Psychophysical Data in R [Internet]. New York, NY: Springer New York; 2012. Available from: https://link.springer.com/10.1007/978-1-4614-4475-6

1. P-values were obtained by using an unequal-variance t-test. [↑](#footnote-ref-1)
